# Mendelian randomization study supports the causal association between serum cystatin C and risk of diabetic nephropathy

**DOI:** 10.3389/fendo.2022.1043174

**Published:** 2022-11-17

**Authors:** Baiyu Feng, Yu Lu, Lin Ye, Lijun Yin, Yingjun Zhou, Anqun Chen

**Affiliations:** ^1^ Department of Nephrology, Hunan Key Laboratory of Kidney Disease and Blood Purification, Institute of Nephrology, The Second Xiangya Hospital at Central South University, Changsha, China; ^2^ Department of Health Sciences, Boston University College of Health and Rehabilitation Sciences: Sargent College, Boston University, Boston, MA, United States

**Keywords:** Mendelian randomization, cystatin C, diabetic nephropathy, biomarker, glomerular filtration rate

## Abstract

**Aims:**

Cystatin C, an inhibitor of cysteine protease, has been used as a biomarker for estimating glomerular filtration rate. However, the causal relation between cystatin C and diabetic nephropathy remains uncertain.

**Methods:**

We assessed the causal effect of cystatin C together with other five serum biomarkers including KIM-1, GDF-15, TBIL, uric acid, and Scr on diabetic nephropathy by Mendelian randomization (MR) analysis. 234 genetic variants were selected as instrumental variables to evaluate the causal effect of cystatin C (N_GWAS_=361194) on diabetic nephropathy (Ncase/Ncontrol up to 3283/210463). Multivariable MR (MVMR) was performed to assess the stability of cystatin C’s causal relationship. Two-step MR was used to assess the mediation effect of BMI and SBP.

**Results:**

Among the six serum biomarkers, only cystatin C causally associated with diabetic nephropathy (IVW OR: 1.36, 95%CI [1.15, 1.61]). After adjusting for the potential confounders BMI and SBP, cystatin C maintained its causal effect on the DN (OR: 1.17, 95%CI [1.02, 1.33]), which means that the risk of DN increased by 17% with an approximate 1 standard deviation (SD) increment of serum cystatin C level. Two-step MR results indicated that BMI might mediate the causal effect of cystatin C on diabetic nephropathy.

**Interpretation:**

Our findings discovered that cystatin C was a risk factor for diabetic nephropathy independent of BMI and SBP in diabetes mellitus patients. Future research is required to illustrate the underlying mechanism and prove targeting circulating cystatin C could be a potential therapy method.

## Introduction

Diabetic nephropathy (DN), a most common complication of diabetes mellitus (DM), is the main causes of end-stage renal disease, and it occurs in 25% to 40% of DM patients worldwide (1, 2). DN is often clinically diagnosed based on persistently increased albuminuria with a ratio of microalbumin and urine creatinine more than 300 mg/g or estimated glomerular filtration rate (eGFR) less than 60 ml/min/1.73m^2^ (3). The therapeutic options for DN were very limited. Therefore, constant search for potential novel therapeutic targets is in big need.

Many risk factors have been recognized to be related with the development and progression of DN in the recent decade (1). Several studies identified plasma kidney injury molecule 1(KIM-1) as a positive predictor of ESRD in T1DM patients and it could predict the early decline of eGFR as well as progression to chronic kidney disease stage 3 without macroalbuminuria (4, 5). Growth differentiation factor-15 (GDF-15) was reported to be a predictor of the rapid deterioration of renal function (6, 7). A recent meta-analysis indicated that total bilirubin level was negatively correlated with the risk of DN (8). Another meta-analysis of 25741 T2DM patients revealed that each increase of 1mg/dl of serum uric acid could increase the risk of DN by 24% (9). Serum creatinine (Scr) and cystatin C are routinely utilized to estimate eGFR. However, a recent study suggested that high level of baseline cystatin C and high velocity of increase of cystatin C in T2DM patients were more likely to develop DN in later life (10). Recent study also reported that high serum creatinine (Scr) variability could independently predict the onset of albuminuria in T2DM patients (11). However, these observational studies couldn’t conclude the causal association between these risk factors and DN.

Mendelian randomization (MR) can explore whether risk factors are causally linked to the outcome by analyzing genetic variants as instrumental variables, which represents with single nucleotide polymorphisms (SNPs). Since the gene randomly distributed at conception, MR can mimic randomized trials and minimize the effect of confounders biasing observational studies (12). Benefit by the recent comprehensive meta-analysis with the GWAS of DN, we performed MR analysis to access the possible causal effect of these risk factors on DN. In this study, we used MR to evaluate whether the following six serum biomarkers of renal function or renal injury (cystatin C, KIM-1, GDF-15, TBIL, uric acid, and Scr) casually associate with DN. Interestingly, it turned out only serum cystatin C was casually linked to DN. Subsequently, we analyzed the SNPs relative to cystatin C and found that the majority of them were related to body mass index (BMI) and systolic blood pressure (SBP). Thus, we further validated the causal relationship between serum cystatin C and DN by multivariable MR using SBP and BMI as confounders, which indicated the slightly alleviated detrimental casual effect. Besides, two-step MR indicated that BMI might play as a mediator between cystatin C and DN. Thus, we concluded that cystatin C was a risk factor in the development of DN independent of BMI and SBP in diabetes mellitus patients.

## Materials and methods

### Overall study design

**Figure 1:**
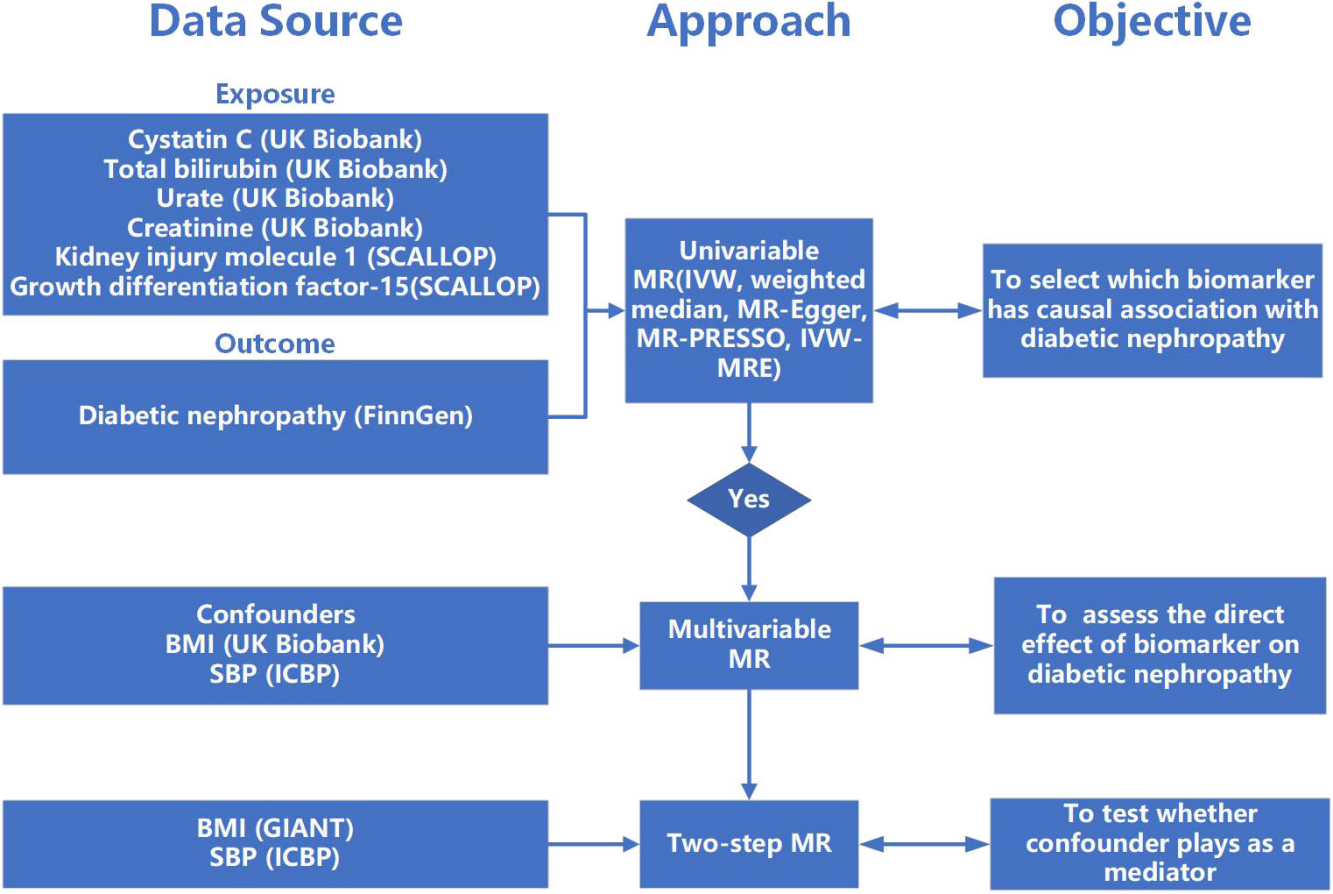
The flow of study. MR, Mendelian randomization; IVW, inverse-variance weighted; MR-PRESSO, Mendelian randomization pleiotropy residual sum and outlier; IVW-MRE, multiplicative random effects inverse variance weighted; BMI, body mass index; SBP, systolic blood pressure.

MR analysis was used to evaluate the causal association between serum biomarkers and diabetic nephropathy, which is based on three assumptions: Assumption 1, the selected genetic variants should be robustly correlated with serum biomarkers; Assumption 2, the genetic variants should not associate with the confounders between the relationship of biomarkers and DN; Assumption 3, the genetic variants should only associate with DN *via* serum biomarkers. Two-sample univariable Mendelian randomization was implemented to evaluate the causal association between multiple serum biomarkers (cystatin C, Scr, urate, total bilirubin, KIM-1, GDF-15) and DN. Biomarkers with significant causal effects on the outcome will be further searched for potential confounders from published articles and Phenoscanner V2 (http://www.phenoscanner.medschl.cam.ac.uk/). Multivariable Mendelian randomization that included biomarkers with significant causal effect and their confounders will be implemented to validate their causal association. Once a causal relationship was established, two-step MR was used to investigate whether confounder plays as a mediator between cystatin C and DN (**Figure 1**).

### Ethics

The summary-level data of GWAS used in this study are publicly accessible, and the original study have acquired ethical approval and informed consent.

### Data source and instrumental variable selection

Instrumental variables (IV) were extracted from GWAS data and SNPs with high linkage disequilibrium were removed. Independent SNP is defined by r^2^ < 0.001 and clumping distance >1Mb using 1,000 genomes reference panel for Europeans (https://www.internationalgenome.org/). Genetic variants that were highly associated with cystatin C, creatinine, urate, and total bilirubin were selected from a GWAS cohort conducted by the Neale Lab Consortium including 361194 individuals of European ancestry (http://www.nealelab.is/uk-biobank). IVs for KIM-1 and GDF-15 were obtained from a meta-analysis including up to 21,758 individuals of European ancestry (13). Genetic variants of confounder SBP were obtained from a GWAS of a meta-analysis that contains over one million European samples from International Consortium for Blood Pressure (ICBP) and UK Biobank (UKB) (14). Genetic variants of confounder BMI were retrieved from GWAS performed by the Neale Lab consortium including 336,107 European individuals (http://www.nealelab.is/uk-biobank). The BMI GWAS used for two-step MR was obtained from the Genetic Investigation of Anthropometric Traits (GIANT) Consortium including 681,275 samples of European ancestry (15).

Diabetic nephropathy as outcome was defined when there was glomerular disorders in the patients with diabetes mellitus with the criterion of ICD-10 (code: N08.3*), summary statistics of which was from FinnGen biobank including 213,746 European individuals (3283 cases and 210463 controls) (16). Except for GDF-15, IVs from other exposures were extracted with a genome-wide significant threshold (p<5E^-8^). Genetic variants of GDF-15 were obtained with a lower threshold (p<1E^-5^) since few IVs were identified with the original threshold. Palindromic SNPs were further excluded from the IV list. For those instruments that are missing in the outcome, proxy SNP with LD score>0.8 was used. In order to satisfy MR assumption three, SNPs with significant association with the outcome were excluded. The F statistic was calculated using the formula: F = beta^2^/se^2^, where beta represents the effect of SNP on the exposure and se is the standard error of the beta, to assess whether there is a possibility of weak instrument bias (17). R^2^ calculated by the following formula: 2 × EAF × (1-EAF) × beta^2^, where EAF represents the effect allele frequency of the SNP, represents the proportion of variance of the exposure explained by SNPs (18).

### Statistical analysis

In this study, inverse variance weighted (IVW) analysis was utilized as the major statistic method. Meanwhile, MR-Egger regression, weighted median, and MR-PRESSO were also performed as complementary methods to validate the IVW result. The IVW method could combine each genetic variant’s Wald estimate in a meta-analysis model and produce unbiased result if horizontal pleiotropy is balanced (19). MR-Egger regression can detect pleiotropy through the intercept it produces while its causal estimate can be largely affected by outliers (20). The weighted median method can provide consistent results even if as many as 50% of instrumental variables are invalid. Mendelian randomization pleiotropy residual sum and outlier (MR-PRESSO) (21) could detect outliers with horizontal pleiotropy (p<0.05), and return a corrected causal estimate after removing them. To validate the robustness of the MR result, Cochran’s Q statistic was performed to detect heterogeneity among instrumental variables. If heterogeneity exists, the multiplicative random effects inverse variance weighted method was further performed (22) to validate the previous MR estimates. Leave-one-out analysis was used to determine whether the SNPs strongly affect the stability of causal estimates.

Multivariable MR (MVMR) was performed to assess the stability of the significant causal relationships, which could estimate the causal relationship between each exposure and a single outcome, producing a causal estimate of direct effect and adjusting pleiotropy caused by other exposures that were included in the MVMR analysis (23). Two-step MR was used to assess the confounder’s mediation effect (24). Firstly, IVs of cystatin C were utilized to perform UVMR analysis against confounder. Secondly, MVMR analysis was used to estimate the causal effect of confounder on DN adjusted for cystatin C. Potential confounders were obtained based on published papers and the online search with Phenoscanner V2. We further calculated the proportion of the mediation effect of confounders by utilizing the product of coefficients method. We first estimated the causal effect of cystatin C on individual confounder, then multiplying the confounder’s effect on DN adjusted for cystatin C, which produced the indirect effect. Finally, we assessed the proportion of mediation effect through dividing the indirect effect by the total effect which in this case is the causal effect of cystatin C on DN. The standard errors were generated by using the delta method. Results were displayed in the form of odds ratio (OR) per an approximate 1 standard deviation (SD) increment. In this study, the statistical power of Mendelian randomization is calculated using mRnd (https://shiny.cnsgenomics.com/mRnd/) with a type 1 error rate of 0.05 (25). Two-sided p<0.05 level of significance is used in all estimates. Statistical analysis was carried out with ‘‘TwosampleMR’’ and ‘‘MR-PRESSO’’ packages in R version 4.1.3.

## Results

### Selection of genetic instrumental variables of exposures

Summary information of selected IVs of 6 exposures was presented in **Table 1**. The mean concentration of cystatin C, creatinine, urate, and total bilirubin obtained from UK biobank’s website which contain the same cohort from GWAS database used in this study but with more participants were 0.908 ± 0.176 mg/L, 72.407 ± 18.524 μmol/L, 309.398 ± 80.394 μmol/L and 9.119 ± 4.425 μmol/L, respectively. The number of IVs varies from 11 to 243, explaining the 4.15% ~ 28.21% variance of corresponding exposure. General F statistics of all exposures and each selected SNP was greater than 10, suggesting that instrumental variables were valid and robust to be included in further MR analysis. The detailed information of all selected SNPs of six exposures was presented in **Tables S1**–**S6**.

**Table 1:** A summary of GWAS summary statistics for six different serum biomarkers.

Exposures	Dataset source	Sample size	NSNP	R2(%)	F
Cystatin C	UK Biobank	361194	234	9.59	163.6084
Total bilirubin	UK Biobank	361194	99	28.21	1433.199
Urate	UK Biobank	361194	188	7.35	152.2644
Creatinine	UK Biobank	361194	243	4.15	64.37972
Kidney injury molecule 1 levels	SCALLOP	21,758	11	13.68	313.1804
Growth differentiation factor-15 levels	SCALLOP	21,758	18	5.67	72.59881

NSNP, the number of SNP included in the MR analysis; R2(%), the proportion of variance explained by included SNPs of each exposure; F, the general F statistic for each biomarker.

### The significant causal effect of serum cystatin C on DN with univariable MR

**Figure 2:**
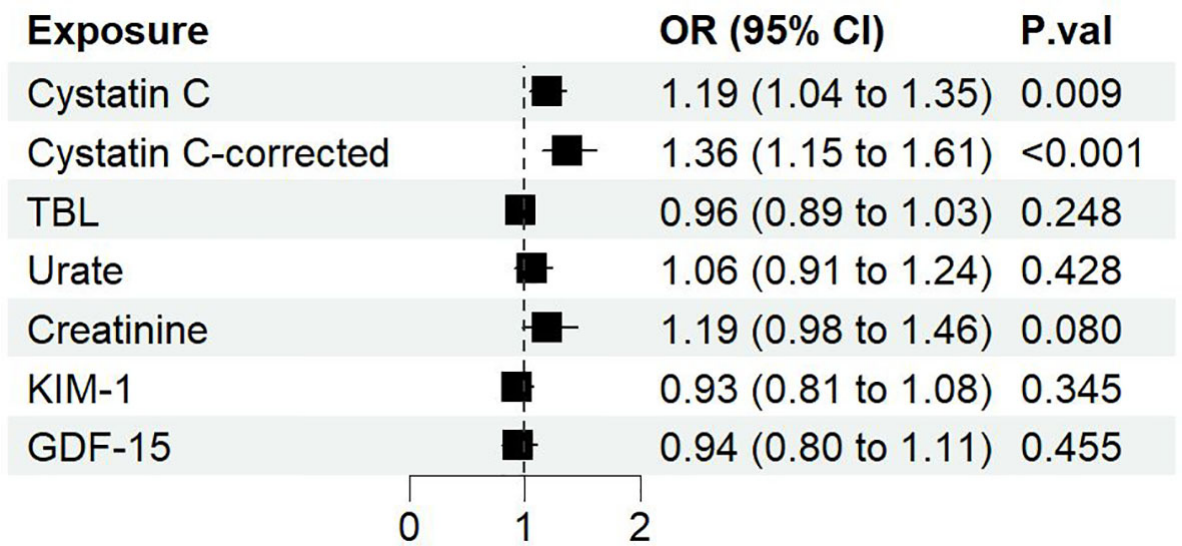
The forest plot of different serum biomarkers’ effect on diabetic nephropathy. OR, odds ratio; CI, confidence interval; P.val, the p-value of IVW MR analysis.

Among 6 serum biomarkers, only cystatin C has a significant causal effect on diabetic nephropathy as a risk factor (IVW OR: 1.19, 95%CI [1.04, 1.35], p=0.009) (**Figure 2**). The same causal direction was observed in MR-Egger, weighted median, and MR-PRESSO analysis (**Figure S1**). Hence, the risk of diabetic nephropathy would increase by 19% with per SD increase of cystatin C. Cochran’s Q test of cystatin C indicates that there is evidence of heterogeneity (IVW p<0.05), while no indication of pleiotropy in MR-Egger (p for intercept>0.05) (**Table S7**). Multiplicative random effects IVW method returned a result resembled to IVW (OR: 1.19, 95%CI [1.04, 1.35], p=0.009) (**Figure S1**). Leave-one-out analysis indicated a similar result to Cochran’s Q-test, suggesting some SNPs might influence the causal estimate. We further identified rs734801, a cystatin C gene (CST3), as a significant IV that could strongly affect the MR result from the leave-one-out analysis (**Figure S2**).

Although horizontal pleiotropy may exist with a p<0.05 as suggested with the global test of MR-PRESSO, the distortion test showed that there is no difference whether the pleiotropic outliers were removed or not (p=0.91). Therefore, we further removed all SNPs identified by MR-PRESSO which may cause horizontal pleiotropy (rs10200647, rs36207014, rs734801, rs77924615, rs80138475). The resulting data showed that cystatin C has a greater causal association with diabetic nephropathy (IVW OR: 1.36, 95%CI [1.15, 1.61], p=0.0004), and Cochran’s Q-test no longer showed evidence of heterogeneity (IVW p>0.05) (**Table S7**). On the basis of the sample size of 213746 individuals (3283 cases and 210463 controls) and setting the explained variance of 4.76%, our study has 99% power to detect effect of serum cystatin C on DN with an OR of 1.36.

### Association of rs734801 in the CST3 gene with cystatin C and DN

As mentioned previously, rs734801 of CST3 gene, which encoded the most plentiful extracellular inhibitor of cysteine proteases (26), strongly affect the MR result based on the leave-one-out analysis. We further examined rs734801, finding that it contributed most to the genetic control of serum cystatin C, explaining 4.7% of the variance. This SNP strongly associated with cystatin C (beta=- 0.37, p<1E^–200^), but not with DN (OR =0.99, p=0.767) (**Table S1**).

### The significant causal effect of serum cystatin C on DN with MVMR

**Figure 3:**
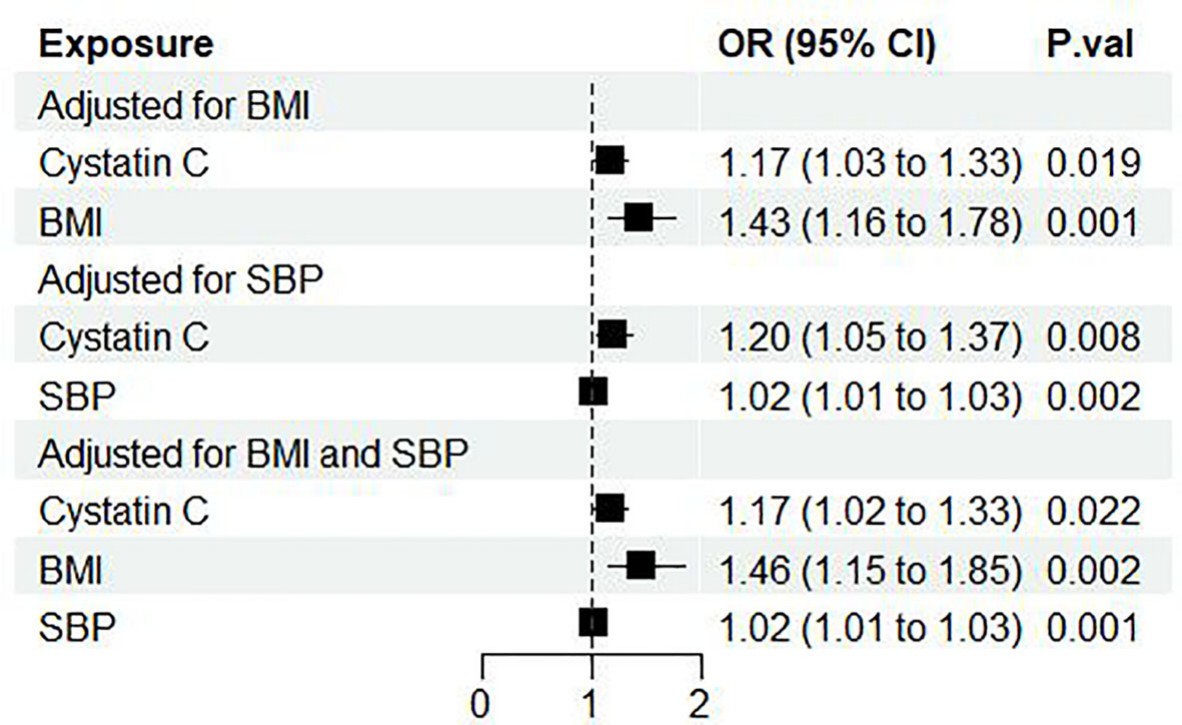
Forest plot of cystatin C’s effect on diabetic nephropathy adjusted for BMI and/or SBP. OR, odds ratio; CI, confidence interval; P.val, the p-value of IVW MR analysis. BMI, body mass index; SBP, systolic blood pressure.

Next, we performed multivariable MR to further analyze the direct effect of cystatin C on diabetic nephropathy. Through online searching IVs selected for serum cystatin C with Phenoscanner V2, we found several potential confounding phenotypes (e.g. BMI, SBP, DBP, log eGFR, hypertension, cholesterol). Finally, we chose BMI and SBP as adjusted confounders in the MR analysis for the following reasons: First, BMI and SBP were the most frequent phenotypes in the process of searching among all the potential confounding phenotypes. Second, BMI and SBP were reported to be risk factors in published MR analysis (27, 28). Subsequently, we implemented three rounds of MVMR: cystatin C against diabetic nephropathy adjusted for (1) BMI alone (2) SBP alone (3) BMI and SBP combined. It showed that cystatin C maintained its causal effect on the outcome no matter adjusted for BMI alone (OR: 1.17, 95%CI [1.03, 1.33], p=0.019), SBP alone (OR: 1.20, 95%CI [1.05, 1.37], p=0.009) or both (OR: 1.17, 95%CI [1.02, 1.33], p=0.02) (**Figure 3**).

### BMI could be a mediator between cystatin C and DN

**Figure 4:**
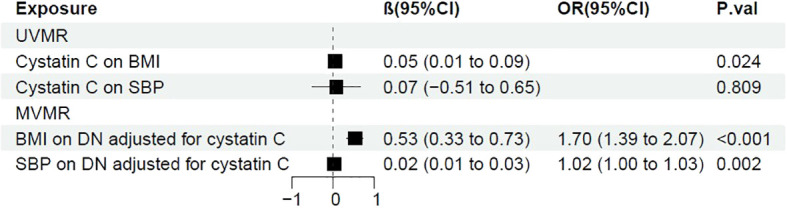
Forest plot of two-step MR with BMI and SBP. OR, odds ratio; CI, confidence interval; P.val, the p-value of IVW MR analysis. BMI, body mass index; SBP, systolic blood pressure.

Next, we performed two-step MR to further investigate whether BMI, SBP functioned as mediator between cystatin C and DN. It showed that there was a casual association between cystatin C and BMI (β=0.05, 95%CI [0.01, 0.09], p=0.024). After adjusted for cystatin C, BMI showed detrimental effect on DN (OR: 1.70, 95%CI [1.39, 2.07], p<0.001). The proportion mediated by BMI was 15.2% (95%CI [4.93%, 22.6%] (**Figure 4**).

Although SBP was previously shown to be a risk factor of DN, we failed to make a conclusion that it played as a mediator between cystatin C and DN, since a causal relationship between cystatin C and SBP was not significant (p=0.81) (**Figure 4**).

## Discussion

In the present study, we took advantage of MR analysis to thoroughly examine the causal association between six serum biomarkers (Cystatin C, KIM-1, GDF-15, TBIL, Urate, and Scr) and DN. Among the six biomarkers, only cystatin C positively associated with the risk of DN. There was no statistical significance for the genetic relationship among the other five biomarkers and DN.

Several studies have shown that high levels of serum urate might cause CKD progression through a mechanism such as excessive production of nitric oxide, activating the renin-angiotensin system, stimulating the proliferation of vascular smooth muscle cell, and obstruction from urate crystals. However, a previous MR study showed that there is no causal relation between serum urate levels and CKD (29) and randomized, controlled trials (RCTs) consistently show that lowering serum urate with allopurinol treatment has no benefits on kidney outcomes among patients with early-to-moderate DN (30). Consistent with this, our results suggest that serum uric acid level didn’t casually link to DN.

Although KIM-1, GDF-15, and TBIL were previously reported to contribute to tubular injury (31, 32) and predict the progression of CKD (4–8). Our data did not show the causally link of these molecules with DN. The reasons of these discrepancies might be as follows: First, the sample sizes reported in the previous studies are too small. Second, most of these studies are observational, which may not reveal a causal relationship but may arise reverse causality because of confounding factors. Third, the experimental results from animal studies may not fully translate into patients with CKD.

Cystatin C, a cysteine protease inhibitor, regulating the activity of cathepsins S and K, have multiple functions in human vascular pathophysiology (33), which is usually used as a measure for GFR. Elevated serum cystatin C routinely serves as an early and sensitive biomarker of impaired renal function (34). Interestingly, our data indicated that serum cystatin C is causally correlated with DN. Additionally, we found that a single SNP (rs734801) in the CST3 gene had a strong association with cystatin C. Besides, we performed another MR analysis excluding this SNP along with another four IVs (rs10200647, rs36207014, rs77924615, rs80138475) which were identified by MR-PRESSO, and obtained a more significant causal effect on DN. Consistent with us, several observational studies demonstrated that cystatin C levels are correlated with the prevalence of T2DM (35) and obesity in adolescents with age of 14-17 years independently of other confounding risk factors (36).

The mechanisms underlying the casual relationship between cystatin C and DN are unclear. There are several potential explanations. Firstly, cystatin C function as a cysteine proteinase inhibitor, which regulated the protease-antiprotease activities of the vascular wall. Thus, the imbalance between cystatin C and cysteine cathepsins might lead to the remodeling of the vascular wall (37). Second, cystatin C was previously reported to be involved in the amplification of cytokines and neuroinflammation in microglia and vascular endothelial injury (38). As inflammation is the hallmark of DN and endothelium injury plays a pivotal role in the occurrence and progression of DN, cystatin C may participate in in the pathological process of DN. Third, cystatin C promotes the proliferation of T cells and differentiation of T cells towards Th1/Th17 cell, which promotes the immune response (39). There is evidence that cystatin C is implicated in several inflammatory autoimmune diseases such as rheumatoid arthritis (40). Since both innate and adaptive immune systems and renal inflammation contribute to the development and progression of DN (41), cystatin C may promote inflammation in DN. However, whether elevated serum cystatin C results in DN progression through endothelium injury, remodeling of vascular wall, and the immune response requires further investigations.

Our study has some limitations: First, since the GWAS derives from European ancestry, generalizability to other ethnicities is limited. Second, due to lacking of individual-level data of GWAS, we cannot explicitly present the baseline data of participants or further stratify serum cystatin C to calculate more detailed causal effect. Third, despite we adjust potential pleiotropy, there is still chance for other confounders to influence the causal estimate. Hence, future research is warranted for further validating our findings.

## Conclusions

Our results suggest that there was a causal relationship between serum cystatin C and DN in diabetic patients, which warns us that cystatin C is not only a biomarker bur also a risk factor for DN progression.

## Data availability statement

Summary statistics are available from each consortium (details in Materials and methods) or via the MR-Base platform (https://gwas.mrcieu.ac.uk/).

## Author contributions

BF and AC designed the study and drafted the manuscript. BF, YL, LY, LJY, YZ, and AC acquired the data, performed statistical analysis and manuscript revision. All authors contributed to the article and approved the submitted version.

## Funding

AC is supported by grants from the National Natural Science Foundation for Excellent Young Scholars (NO. 82222013) and Natural Science Foundation for Distinguished Young Scholars of Hunan province (2021JJ10075).

## Conflict of interest

The authors declare that the research was conducted in the absence of any commercial or financial relationships that could be construed as a potential conflict of interest.

## Publisher’s note

All claims expressed in this article are solely those of the authors and do not necessarily represent those of their affiliated organizations, or those of the publisher, the editors and the reviewers. Any product that may be evaluated in this article, or claim that may be made by its manufacturer, is not guaranteed or endorsed by the publisher.
