# Supplementary material for: Mendelian randomization study supports the causal association between serum cystatin C and risk of diabetic nephropathy
Source: Front Endocrinol (Lausanne). 2022 Nov 17;13:1043174. doi: 10.3389/fendo.2022.1043174 (PMC9724588; doi:10.3389/fendo.2022.1043174)
Supplement: Supplementary file 1 [file Table_1.docx]

**Supplementary Table 1**: Instrumental variables of cystatin C. SNP, the rsID of genetic variants; A1, the effect allele; A2, the other allele; Beta, the effect size of A1 on the exposure; Se, the standard error of beta; Proxy, the proxy SNP in the outcome; P, the p-value of beta; R2, the proportion of variance explained by each SNP; F, the F statistic

| SNP | A1 | A2 | Beta | EAF | Proxy | P | Se | R2 | F |
| --- | --- | --- | --- | --- | --- | --- | --- | --- | --- |
| rs1000423 | T | C | -0.0177 | 0.73573 |  | 6.92E-13 | 0.00247 | 0.00012 | 51.5738 |
| rs10009166 | A | C | 0.0166 | 0.33351 |  | 7.19E-13 | 0.00231 | 0.00012 | 51.4917 |
| rs10051765 | C | T | 0.0451 | 0.33226 |  | 6.27E-85 | 0.00231 | 0.0009 | 381.576 |
| rs10109414 | T | C | 0.04294 | 0.41516 |  | 2.95E-84 | 0.00221 | 0.0009 | 378.494 |
| rs1011731 | A | G | 0.01702 | 0.5667 |  | 9.61E-15 | 0.0022 | 0.00014 | 59.9796 |
| rs10160464 | G | A | -0.0155 | 0.32401 |  | 2.19E-11 | 0.00232 | 0.00011 | 44.7978 |
| rs10180164 | C | A | 0.04806 | 0.0322 |  | 6.76E-15 | 0.00617 | 0.00014 | 60.6749 |
| rs10189685 | A | G | 0.03774 | 0.29438 |  | 1.95E-56 | 0.00238 | 0.00059 | 250.648 |
| rs10200647 | C | T | -0.0166 | 0.24536 |  | 5.67E-11 | 0.00253 | 0.0001 | 42.9349 |
| rs10211596 | A | G | 0.01622 | 0.5328 |  | 1.22E-13 | 0.00219 | 0.00013 | 54.9902 |
| rs102274 | C | T | -0.0186 | 0.34658 |  | 4.07E-16 | 0.00229 | 0.00016 | 66.211 |
| rs10265221 | C | T | 0.04988 | 0.28718 | rs10224002 | 1.17E-95 | 0.0024 | 0.00102 | 430.938 |
| rs10437886 | T | C | -0.013 | 0.44921 |  | 3.91E-09 | 0.0022 | 8.3E-05 | 34.669 |
| rs1047891 | A | C | -0.0259 | 0.31574 |  | 1.75E-28 | 0.00234 | 0.00029 | 122.569 |
| rs1058056 | G | A | -0.0171 | 0.83341 |  | 4.96E-09 | 0.00292 | 8.1E-05 | 34.2067 |
| rs10746942 | A | G | -0.0234 | 0.62167 |  | 1.87E-25 | 0.00225 | 0.00026 | 108.742 |
| rs10821907 | T | C | -0.0247 | 0.17899 |  | 9.37E-18 | 0.00287 | 0.00018 | 73.6502 |
| rs10846156 | G | T | -0.0298 | 0.202 | rs10846157 | 5.92E-28 | 0.00272 | 0.00029 | 120.148 |
| rs10875290 | T | G | 0.01753 | 0.39176 |  | 4.32E-15 | 0.00223 | 0.00015 | 61.551 |
| rs10911678 | C | T | -0.0153 | 0.41114 |  | 3.96E-12 | 0.00221 | 0.00011 | 48.1455 |
| rs10912860 | G | A | -0.0156 | 0.2276 |  | 2.20E-09 | 0.0026 | 8.5E-05 | 35.7871 |
| rs10933149 | T | C | 0.0134 | 0.31315 |  | 1.28E-08 | 0.00236 | 7.7E-05 | 32.3653 |
| rs10953356 | G | A | 0.02911 | 0.29015 |  | 6.15E-34 | 0.0024 | 0.00035 | 147.526 |
| rs1099455 | A | G | -0.0704 | 0.07478 |  | 9.21E-65 | 0.00414 | 0.00069 | 288.898 |
| rs11000980 | G | A | -0.0178 | 0.42213 |  | 8.30E-16 | 0.00221 | 0.00015 | 64.8065 |
| rs11071726 | C | T | -0.0227 | 0.64736 |  | 2.31E-23 | 0.00228 | 0.00024 | 99.187 |
| rs11072567 | G | A | 0.03676 | 0.51212 |  | 5.80E-64 | 0.00218 | 0.00068 | 285.214 |
| rs11082431 | T | C | 0.01435 | 0.30274 |  | 1.49E-09 | 0.00237 | 8.7E-05 | 36.5493 |
| rs11115974 | A | G | 0.016 | 0.55011 |  | 3.08E-13 | 0.00219 | 0.00013 | 53.1616 |
| rs111622274 | T | C | -0.0353 | 0.03196 | rs74886619 | 1.71E-08 | 0.00627 | 7.7E-05 | 31.7987 |
| rs11227279 | A | G | 0.02969 | 0.3449 |  | 1.53E-38 | 0.00229 | 0.0004 | 168.594 |
| rs113243210 | T | C | -0.018 | 0.14109 |  | 1.14E-08 | 0.00315 | 7.8E-05 | 32.5872 |
| rs113367286 | T | C | -0.0148 | 0.27933 |  | 1.29E-09 | 0.00243 | 8.8E-05 | 36.823 |
| rs1135498 | C | T | -0.0172 | 0.17284 |  | 2.66E-08 | 0.00309 | 8.4E-05 | 30.9419 |
| rs114388677 | A | G | 0.03496 | 0.03247 |  | 2.28E-08 | 0.00625 | 7.7E-05 | 31.2394 |
| rs114612307 | T | C | -0.0484 | 0.01622 |  | 1.96E-08 | 0.00862 | 7.5E-05 | 31.5338 |
| rs11557049 | T | C | 0.0296 | 0.06678 |  | 1.15E-11 | 0.00436 | 0.00011 | 46.0566 |
| rs1158127 | G | A | 0.02417 | 0.8958 |  | 2.76E-10 | 0.00383 | 0.00011 | 39.8357 |
| rs11616030 | C | A | -0.0308 | 0.08702 |  | 2.21E-15 | 0.00388 | 0.00015 | 62.8758 |
| rs11629006 | C | T | 0.02108 | 0.18161 |  | 7.97E-14 | 0.00282 | 0.00013 | 55.8216 |
| rs11645023 | A | G | -0.0189 | 0.23425 |  | 1.87E-13 | 0.00257 | 0.00013 | 54.1376 |
| rs11651885 | A | G | 0.02944 | 0.78196 |  | 7.79E-29 | 0.00264 | 0.0003 | 124.178 |
| rs1169300 | A | G | 0.03169 | 0.29077 |  | 1.01E-39 | 0.0024 | 0.00041 | 174.001 |
| rs117081694 | A | G | -0.0335 | 0.03452 |  | 1.95E-08 | 0.00596 | 7.5E-05 | 31.5455 |
| rs11795343 | C | T | -0.0124 | 0.40114 |  | 2.56E-08 | 0.00223 | 7.4E-05 | 31.0172 |
| rs12023563 | A | G | -0.0146 | 0.29232 |  | 1.19E-09 | 0.0024 | 8.8E-05 | 36.9808 |
| rs12108134 | T | C | 0.01446 | 0.40517 |  | 6.83E-11 | 0.00222 | 0.0001 | 42.572 |
| rs12367963 | T | C | 0.02476 | 0.09037 |  | 7.82E-11 | 0.00381 | 0.0001 | 42.306 |
| rs12476256 | C | T | -0.0148 | 0.58127 |  | 2.22E-11 | 0.00222 | 0.00011 | 44.7721 |
| rs12596890 | G | A | 0.01694 | 0.20135 |  | 4.60E-10 | 0.00272 | 9.2E-05 | 38.8386 |
| rs1260326 | C | T | 0.02174 | 0.6069 |  | 1.61E-22 | 0.00223 | 0.00023 | 95.3491 |
| rs12651794 | C | T | -0.0146 | 0.29296 |  | 1.34E-09 | 0.0024 | 8.8E-05 | 36.7495 |
| rs1265842 | C | T | -0.0161 | 0.51909 |  | 1.73E-13 | 0.00219 | 0.00013 | 54.2947 |
| rs12713261 | C | T | 0.01723 | 0.35211 |  | 6.62E-14 | 0.0023 | 0.00014 | 56.1848 |
| rs12736181 | A | G | 0.01784 | 0.31461 |  | 2.56E-14 | 0.00234 | 0.00014 | 58.057 |
| rs12751255 | C | A | -0.0166 | 0.36524 |  | 2.04E-13 | 0.00226 | 0.00013 | 53.9729 |
| rs12753566 | C | T | 0.01757 | 0.29199 |  | 2.82E-13 | 0.00241 | 0.00013 | 53.3327 |
| rs12883201 | T | C | -0.0177 | 0.23684 |  | 6.55E-12 | 0.00257 | 0.00011 | 47.1601 |
| rs13000374 | G | A | -0.0156 | 0.72962 |  | 2.53E-10 | 0.00246 | 9.5E-05 | 40.014 |
| rs13044896 | C | T | -0.0451 | 0.08418 |  | 1.26E-28 | 0.00406 | 0.00031 | 123.221 |
| rs13119468 | T | C | -0.0153 | 0.2088 |  | 1.26E-08 | 0.00269 | 7.8E-05 | 32.4 |
| rs13159523 | G | A | -0.0139 | 0.47987 |  | 3.10E-10 | 0.00221 | 9.7E-05 | 39.6084 |
| rs1317983 | C | T | 0.03717 | 0.69385 |  | 5.59E-56 | 0.00236 | 0.00059 | 248.568 |
| rs13281719 | T | C | -0.0213 | 0.0916 |  | 2.23E-08 | 0.0038 | 7.5E-05 | 31.2826 |
| rs13329240 | A | G | 0.01938 | 0.31722 |  | 1.17E-16 | 0.00234 | 0.00016 | 68.6709 |
| rs13391258 | T | C | -0.0266 | 0.2289 |  | 1.06E-24 | 0.00259 | 0.00025 | 105.289 |
| rs141262170 | G | A | -0.0523 | 0.04014 |  | 8.98E-21 | 0.0056 | 0.00021 | 87.3868 |
| rs141350020 | T | C | -0.0369 | 0.04227 |  | 2.98E-11 | 0.00555 | 0.00011 | 44.1907 |
| rs1458038 | T | C | -0.0156 | 0.29292 |  | 9.33E-11 | 0.0024 | 0.0001 | 41.9586 |
| rs145868254 | T | C | 0.03045 | 0.04935 |  | 1.96E-09 | 0.00507 | 8.7E-05 | 36.0189 |
| rs146828372 | C | A | -0.0205 | 0.13224 | rs56737784 | 5.84E-10 | 0.00331 | 9.7E-05 | 38.3775 |
| rs148185902 | A | G | 0.09496 | 0.0138 |  | 6.15E-22 | 0.00986 | 0.00025 | 92.6909 |
| rs151245 | T | G | 0.01612 | 0.60183 |  | 4.55E-13 | 0.00223 | 0.00012 | 52.3932 |
| rs1554447 | T | C | -0.0191 | 0.85226 |  | 5.77E-10 | 0.00308 | 9.2E-05 | 38.3983 |
| rs1556894 | G | A | -0.0136 | 0.44093 |  | 6.54E-10 | 0.00219 | 9.1E-05 | 38.1526 |
| rs1585499 | T | C | 0.02412 | 0.46143 |  | 3.67E-28 | 0.00219 | 0.00029 | 121.104 |
| rs1681725 | G | A | 0.01201 | 0.50025 |  | 3.74E-08 | 0.00218 | 7.2E-05 | 30.283 |
| rs16853637 | A | G | 0.02219 | 0.12312 |  | 2.11E-11 | 0.00331 | 0.00011 | 44.8735 |
| rs1700820 | G | A | -0.0133 | 0.58874 |  | 1.97E-09 | 0.00221 | 8.5E-05 | 35.9989 |
| rs17037425 | A | G | 0.01989 | 0.1489 |  | 7.74E-11 | 0.00306 | 0.0001 | 42.3272 |
| rs1713806 | A | C | 0.01506 | 0.74222 |  | 1.59E-09 | 0.0025 | 8.7E-05 | 36.4206 |
| rs17184086 | A | G | -0.0529 | 0.02198 |  | 1.19E-12 | 0.00744 | 0.00012 | 50.5147 |
| rs17597926 | A | G | -0.0483 | 0.02553 |  | 2.53E-12 | 0.0069 | 0.00012 | 49.0272 |
| rs17641524 | T | C | 0.01614 | 0.20985 |  | 1.64E-09 | 0.00268 | 8.6E-05 | 36.3651 |
| rs17643734 | G | A | 0.03244 | 0.05663 |  | 5.49E-12 | 0.00471 | 0.00011 | 47.5074 |
| rs1769798 | G | A | 0.01309 | 0.42733 |  | 2.70E-09 | 0.0022 | 8.4E-05 | 35.3907 |
| rs1772973 | G | A | -0.0175 | 0.72645 |  | 8.80E-13 | 0.00245 | 0.00012 | 51.1022 |
| rs17767383 | A | G | 0.0237 | 0.31579 |  | 4.00E-24 | 0.00234 | 0.00024 | 102.669 |
| rs1800961 | T | C | -0.0379 | 0.03143 |  | 1.25E-09 | 0.00624 | 8.7E-05 | 36.8958 |
| rs1852731 | G | A | -0.021 | 0.10346 |  | 4.77E-09 | 0.00358 | 8.2E-05 | 34.285 |
| rs1885800 | T | C | -0.0168 | 0.18142 |  | 2.64E-09 | 0.00283 | 8.4E-05 | 35.4339 |
| rs1895460 | C | A | -0.0131 | 0.52387 |  | 1.87E-09 | 0.00219 | 8.6E-05 | 36.1044 |
| rs198325 | T | C | -0.0308 | 0.22133 |  | 8.45E-32 | 0.00262 | 0.00033 | 137.732 |
| rs1999032 | C | T | -0.0137 | 0.462 |  | 5.45E-10 | 0.0022 | 9.3E-05 | 38.5127 |
| rs2014520 | A | G | -0.0175 | 0.41981 |  | 3.73E-15 | 0.00223 | 0.00015 | 61.8401 |
| rs2049019 | A | C | -0.0183 | 0.68689 |  | 8.87E-15 | 0.00235 | 0.00014 | 60.1354 |
| rs2068888 | A | G | 0.01968 | 0.44894 |  | 2.29E-19 | 0.00219 | 0.00019 | 80.9811 |
| rs2138385 | C | T | 0.02164 | 0.8782 |  | 1.80E-10 | 0.00339 | 0.0001 | 40.67 |
| rs2161796 | C | T | -0.0132 | 0.37514 | rs1518139 | 5.01E-09 | 0.00225 | 8.1E-05 | 34.1888 |
| rs2177556 | G | A | 0.02909 | 0.91545 |  | 1.36E-13 | 0.00393 | 0.00013 | 54.7713 |
| rs2237668 | G | A | -0.0226 | 0.22824 |  | 2.76E-18 | 0.00259 | 0.00018 | 76.0592 |
| rs224143 | A | G | -0.0183 | 0.60142 |  | 2.07E-16 | 0.00223 | 0.00016 | 67.5456 |
| rs2255293 | C | T | -0.0155 | 0.79091 |  | 7.73E-09 | 0.00269 | 8E-05 | 33.3446 |
| rs2282718 | A | G | -0.0127 | 0.37061 |  | 2.30E-08 | 0.00228 | 7.6E-05 | 31.2233 |
| rs2306623 | C | T | -0.0197 | 0.6647 |  | 1.36E-17 | 0.00231 | 0.00017 | 72.9098 |
| rs2306899 | T | C | -0.0143 | 0.2442 |  | 1.71E-08 | 0.00253 | 7.5E-05 | 31.8043 |
| rs2307111 | C | T | -0.0153 | 0.39285 |  | 6.35E-12 | 0.00223 | 0.00011 | 47.219 |
| rs2403966 | T | G | 0.01677 | 0.3038 |  | 1.46E-12 | 0.00237 | 0.00012 | 50.1103 |
| rs2636321 | G | A | -0.0153 | 0.52874 |  | 3.15E-12 | 0.00219 | 0.00012 | 48.5983 |
| rs2756176 | T | C | -0.0143 | 0.39194 |  | 1.33E-10 | 0.00223 | 9.8E-05 | 41.2676 |
| rs2823139 | A | G | 0.0221 | 0.33845 |  | 1.15E-21 | 0.00231 | 0.00022 | 91.4495 |
| rs2834320 | G | A | 0.03056 | 0.16194 |  | 6.96E-25 | 0.00297 | 0.00025 | 106.132 |
| rs28730759 | T | C | -0.0215 | 0.14443 |  | 5.03E-12 | 0.00311 | 0.00011 | 47.6813 |
| rs288762 | C | T | 0.02841 | 0.63674 |  | 6.05E-36 | 0.00227 | 0.00037 | 156.699 |
| rs28929474 | T | C | 0.06711 | 0.0203 |  | 3.72E-18 | 0.00773 | 0.00018 | 75.4705 |
| rs28930677 | T | C | 0.06659 | 0.04697 |  | 2.53E-38 | 0.00514 | 0.0004 | 167.599 |
| rs2899297 | A | G | 0.01252 | 0.59975 |  | 1.76E-08 | 0.00222 | 7.5E-05 | 31.7423 |
| rs2910686 | C | T | -0.016 | 0.44713 |  | 2.77E-13 | 0.00219 | 0.00013 | 53.3729 |
| rs2963033 | C | A | 0.01418 | 0.55356 |  | 2.19E-10 | 0.00223 | 9.9E-05 | 40.292 |
| rs301806 | T | C | 0.01311 | 0.58418 |  | 2.94E-09 | 0.00221 | 8.4E-05 | 35.2242 |
| rs3130266 | A | G | 0.01326 | 0.50284 |  | 1.10E-09 | 0.00218 | 8.8E-05 | 37.1394 |
| rs3184504 | C | T | -0.0608 | 0.51795 |  | 1.38E-171 | 0.00218 | 0.00185 | 780.591 |
| rs3197999 | A | G | -0.0194 | 0.28511 |  | 7.70E-16 | 0.00241 | 0.00015 | 64.9519 |
| rs333947 | A | G | 0.0319 | 0.14951 |  | 1.40E-25 | 0.00305 | 0.00026 | 109.318 |
| rs340029 | T | C | 0.01398 | 0.62092 |  | 4.90E-10 | 0.00225 | 9.2E-05 | 38.7196 |
| rs34517439 | A | C | 0.03553 | 0.12461 |  | 2.27E-26 | 0.00334 | 0.00028 | 112.916 |
| rs35624969 | C | T | 0.01972 | 0.30637 | rs12540011 | 2.95E-16 | 0.00241 | 0.00017 | 66.8423 |
| rs36207014 | A | G | 0.02236 | 0.36721 |  | 7.12E-23 | 0.00227 | 0.00023 | 96.9664 |
| rs3729965 | G | A | -0.0146 | 0.75688 |  | 1.75E-08 | 0.00258 | 7.8E-05 | 31.7569 |
| rs3736905 | G | A | 0.02105 | 0.64857 |  | 2.94E-20 | 0.00228 | 0.0002 | 85.0429 |
| rs3740685 | T | C | 0.01682 | 0.70887 |  | 2.25E-12 | 0.0024 | 0.00012 | 49.2562 |
| rs3769869 | G | A | 0.01957 | 0.19554 |  | 1.12E-12 | 0.00275 | 0.00012 | 50.6265 |
| rs3789688 | C | T | 0.01307 | 0.57117 |  | 3.17E-09 | 0.00221 | 8.4E-05 | 35.0748 |
| rs3888879 | T | C | -0.0227 | 0.34125 |  | 6.67E-23 | 0.0023 | 0.00023 | 97.0947 |
| rs3925584 | C | T | -0.0352 | 0.45458 |  | 1.70E-58 | 0.00218 | 0.00061 | 260.12 |
| rs410335 | C | T | -0.0141 | 0.58767 |  | 1.87E-10 | 0.00221 | 9.6E-05 | 40.6 |
| rs41276588 | A | G | 0.01836 | 0.28693 |  | 3.26E-14 | 0.00242 | 0.00014 | 57.5769 |
| rs4358800 | T | C | 0.01763 | 0.66553 |  | 1.05E-13 | 0.00237 | 0.00014 | 55.2813 |
| rs4442348 | G | A | 0.01536 | 0.49879 |  | 1.99E-12 | 0.00218 | 0.00012 | 49.4963 |
| rs4666821 | T | G | -0.0136 | 0.51211 |  | 4.18E-10 | 0.00218 | 9.2E-05 | 39.0259 |
| rs4771989 | C | A | 0.01302 | 0.66913 |  | 2.07E-08 | 0.00232 | 7.5E-05 | 31.4306 |
| rs4820483 | A | C | 0.01369 | 0.45892 |  | 4.03E-10 | 0.00219 | 9.3E-05 | 39.0975 |
| rs4834191 | T | C | 0.0169 | 0.57877 | rs1372965 | 2.51E-14 | 0.00222 | 0.00014 | 58.0859 |
| rs4836732 | T | C | -0.0138 | 0.52824 |  | 3.07E-10 | 0.00219 | 9.5E-05 | 39.6287 |
| rs4859682 | A | C | 0.06667 | 0.45682 |  | 1.00E-200 | 0.00218 | 0.00221 | 936.012 |
| rs4898541 | A | G | -0.0205 | 0.17388 |  | 1.66E-12 | 0.0029 | 0.00012 | 49.8588 |
| rs4946137 | A | G | 0.01588 | 0.40022 |  | 9.18E-13 | 0.00222 | 0.00012 | 51.0177 |
| rs4959168 | T | C | -0.0188 | 0.62628 |  | 7.79E-17 | 0.00225 | 0.00017 | 69.4716 |
| rs4966019 | T | C | -0.0374 | 0.64051 |  | 5.77E-61 | 0.00227 | 0.00065 | 271.438 |
| rs4968248 | A | G | -0.0123 | 0.60667 |  | 3.94E-08 | 0.00223 | 7.2E-05 | 30.1793 |
| rs4970460 | A | C | -0.0145 | 0.21827 |  | 4.06E-08 | 0.00264 | 7.2E-05 | 30.1227 |
| rs498010 | T | C | -0.019 | 0.18342 |  | 2.40E-11 | 0.00284 | 0.00011 | 44.6135 |
| rs4981832 | T | C | 0.01452 | 0.59234 |  | 6.62E-11 | 0.00222 | 0.0001 | 42.6306 |
| rs5029979 | A | G | 0.0161 | 0.39206 |  | 4.87E-13 | 0.00223 | 0.00012 | 52.2657 |
| rs55887858 | T | G | 0.01381 | 0.26861 |  | 2.19E-08 | 0.00247 | 7.5E-05 | 31.3166 |
| rs55938024 | A | G | 0.0337 | 0.10753 |  | 7.63E-22 | 0.00351 | 0.00022 | 92.2653 |
| rs56376587 | C | A | 0.02459 | 0.48835 |  | 1.04E-28 | 0.00221 | 0.0003 | 123.599 |
| rs569502 | C | T | -0.0168 | 0.66085 |  | 2.71E-13 | 0.0023 | 0.00013 | 53.4141 |
| rs5756822 | T | C | 0.01517 | 0.55877 |  | 4.43E-12 | 0.00219 | 0.00011 | 47.9329 |
| rs57602436 | A | G | 0.01679 | 0.67061 |  | 4.55E-13 | 0.00232 | 0.00012 | 52.3954 |
| rs6029394 | T | G | -0.0138 | 0.48529 |  | 3.02E-10 | 0.00218 | 9.4E-05 | 39.6579 |
| rs60385825 | C | T | 0.0253 | 0.28949 |  | 3.91E-25 | 0.00244 | 0.00026 | 107.275 |
| rs60767324 | C | T | 0.0281 | 0.08669 |  | 4.40E-13 | 0.00388 | 0.00012 | 52.4615 |
| rs6083315 | A | G | -0.0447 | 0.67403 |  | 1.05E-77 | 0.00239 | 0.00088 | 348.358 |
| rs6113840 | T | G | 0.01772 | 0.16424 |  | 1.91E-09 | 0.00295 | 8.6E-05 | 36.0626 |
| rs62033400 | G | A | 0.02062 | 0.3935 |  | 2.38E-20 | 0.00223 | 0.0002 | 85.4524 |
| rs62106258 | C | T | -0.0372 | 0.04834 |  | 2.34E-13 | 0.00508 | 0.00013 | 53.7004 |
| rs621634 | T | G | -0.0147 | 0.26046 |  | 4.48E-09 | 0.0025 | 8.3E-05 | 34.4048 |
| rs62435145 | T | G | 0.03814 | 0.69191 |  | 2.01E-55 | 0.00243 | 0.00062 | 246.009 |
| rs62618693 | T | C | -0.0411 | 0.04537 |  | 3.24E-15 | 0.00522 | 0.00015 | 62.1183 |
| rs6458867 | G | A | 0.01712 | 0.64455 |  | 5.39E-14 | 0.00228 | 0.00013 | 56.5883 |
| rs6532771 | G | A | 0.01489 | 0.25682 |  | 3.48E-09 | 0.00252 | 8.5E-05 | 34.9001 |
| rs6744911 | G | A | -0.0147 | 0.2477 |  | 5.38E-09 | 0.00252 | 8.1E-05 | 34.0498 |
| rs6777400 | A | G | -0.0178 | 0.2586 |  | 9.31E-13 | 0.00249 | 0.00012 | 50.9844 |
| rs6787409 | C | T | -0.0246 | 0.33009 |  | 8.22E-26 | 0.00234 | 0.00027 | 110.361 |
| rs68033110 | A | G | 0.02016 | 0.24697 |  | 3.66E-15 | 0.00256 | 0.00015 | 61.8758 |
| rs6807915 | C | T | 0.01274 | 0.64264 |  | 2.29E-08 | 0.00228 | 7.5E-05 | 31.2324 |
| rs6857 | T | C | -0.0297 | 0.17175 |  | 8.86E-25 | 0.00289 | 0.00025 | 105.654 |
| rs687467 | G | A | 0.02974 | 0.75691 |  | 3.18E-31 | 0.00256 | 0.00033 | 135.104 |
| rs687914 | T | G | 0.0199 | 0.25182 |  | 2.84E-15 | 0.00252 | 0.00015 | 62.38 |
| rs700750 | A | C | 0.02717 | 0.6289 |  | 1.60E-33 | 0.00225 | 0.00034 | 145.61 |
| rs710177 | G | A | 0.01389 | 0.38743 |  | 9.68E-10 | 0.00227 | 9.2E-05 | 37.3908 |
| rs7145592 | T | G | 0.0164 | 0.28825 |  | 9.17E-12 | 0.0024 | 0.00011 | 46.5049 |
| rs7161833 | A | C | -0.0189 | 0.4282 |  | 8.56E-18 | 0.0022 | 0.00017 | 73.8318 |
| rs7207304 | A | G | -0.0201 | 0.28479 | rs7208664 | 1.04E-16 | 0.00242 | 0.00016 | 68.9028 |
| rs7258309 | G | T | -0.0187 | 0.20664 |  | 4.36E-12 | 0.0027 | 0.00011 | 47.9594 |
| rs72747610 | G | A | -0.0153 | 0.24083 |  | 1.93E-09 | 0.00255 | 8.6E-05 | 36.0452 |
| rs72821069 | C | T | 0.01964 | 0.16357 |  | 4.25E-11 | 0.00298 | 0.00011 | 43.4975 |
| rs728538 | G | T | 0.02538 | 0.16689 |  | 5.28E-18 | 0.00293 | 0.00018 | 74.7854 |
| rs7305251 | A | G | 0.01551 | 0.17896 |  | 4.95E-08 | 0.00284 | 7.1E-05 | 29.7371 |
| rs73102389 | A | C | 0.12264 | 0.17541 |  | 1.00E-200 | 0.00285 | 0.00435 | 1850.42 |
| rs73232580 | C | T | -0.0172 | 0.16208 |  | 1.20E-08 | 0.00302 | 8.1E-05 | 32.4955 |
| rs73260458 | T | C | -0.0312 | 0.04731 |  | 1.14E-09 | 0.00513 | 8.8E-05 | 37.0642 |
| rs734801 | G | A | -0.3668 | 0.2231 |  | 1.00E-200 | 0.00255 | 0.04664 | 20664.4 |
| rs7459014 | A | C | -0.0178 | 0.27617 |  | 9.96E-13 | 0.0025 | 0.00013 | 50.8521 |
| rs7490694 | G | A | -0.0251 | 0.71057 |  | 3.08E-25 | 0.00241 | 0.00026 | 107.753 |
| rs7502669 | G | A | -0.0156 | 0.589 |  | 2.87E-12 | 0.00224 | 0.00012 | 48.7756 |
| rs759219 | T | C | -0.0122 | 0.44565 |  | 3.31E-08 | 0.00221 | 7.4E-05 | 30.5135 |
| rs7593570 | A | G | 0.01769 | 0.49293 |  | 4.87E-16 | 0.00218 | 0.00016 | 65.8559 |
| rs76428106 | C | T | 0.0734 | 0.01313 |  | 1.54E-13 | 0.00994 | 0.00014 | 54.5239 |
| rs76433917 | T | C | -0.0355 | 0.03183 |  | 9.90E-09 | 0.0062 | 7.8E-05 | 32.8619 |
| rs7645663 | T | C | -0.0125 | 0.36255 |  | 3.81E-08 | 0.00226 | 7.2E-05 | 30.2454 |
| rs7684939 | A | G | -0.0196 | 0.49097 |  | 2.59E-19 | 0.00218 | 0.00019 | 80.7447 |
| rs7763581 | G | T | -0.0123 | 0.48499 |  | 1.40E-08 | 0.00218 | 7.6E-05 | 32.1931 |
| rs7766720 | C | T | 0.02499 | 0.0782 |  | 7.23E-10 | 0.00406 | 9E-05 | 37.9604 |
| rs77871802 | G | A | -0.0328 | 0.03439 |  | 4.11E-08 | 0.00598 | 7.2E-05 | 30.0969 |
| rs77924615 | A | G | -0.0604 | 0.19766 |  | 2.18E-106 | 0.00276 | 0.00116 | 480.287 |
| rs78040893 | T | G | -0.0349 | 0.05335 |  | 8.39E-13 | 0.00488 | 0.00012 | 51.1949 |
| rs78128878 | C | T | 0.02452 | 0.07256 |  | 5.17E-09 | 0.0042 | 8.1E-05 | 34.1247 |
| rs78260152 | T | C | 0.04159 | 0.08097 |  | 3.90E-25 | 0.00402 | 0.00026 | 107.28 |
| rs78597273 | A | G | -0.0135 | 0.33922 |  | 7.36E-09 | 0.00233 | 8.2E-05 | 33.4381 |
| rs786913 | G | A | -0.0185 | 0.6144 |  | 1.18E-16 | 0.00223 | 0.00016 | 68.6533 |
| rs78691875 | A | C | -0.0481 | 0.02132 |  | 1.57E-10 | 0.00752 | 9.7E-05 | 40.9455 |
| rs7911018 | T | C | 0.01433 | 0.6083 |  | 1.34E-10 | 0.00223 | 9.8E-05 | 41.2582 |
| rs7919595 | G | A | -0.016 | 0.2035 |  | 3.88E-09 | 0.00271 | 8.3E-05 | 34.689 |
| rs7944897 | G | A | 0.01891 | 0.46442 |  | 4.39E-18 | 0.00218 | 0.00018 | 75.1445 |
| rs7952141 | A | G | -0.0191 | 0.38619 |  | 1.93E-17 | 0.00224 | 0.00017 | 72.2166 |
| rs80138475 | T | C | -0.0282 | 0.11295 |  | 3.29E-16 | 0.00345 | 0.00016 | 66.6263 |
| rs8023580 | C | T | 0.01359 | 0.2744 |  | 2.64E-08 | 0.00244 | 7.4E-05 | 30.9527 |
| rs8058761 | A | G | 0.01338 | 0.32847 |  | 1.50E-08 | 0.00236 | 7.9E-05 | 32.0582 |
| rs807624 | T | G | -0.033 | 0.35772 |  | 4.73E-48 | 0.00227 | 0.0005 | 212.19 |
| rs8101667 | C | T | 0.03002 | 0.66112 |  | 6.46E-39 | 0.0023 | 0.0004 | 170.308 |
| rs835223 | T | C | 0.02122 | 0.41092 |  | 1.13E-21 | 0.00222 | 0.00022 | 91.4889 |
| rs854917 | T | C | -0.0183 | 0.7376 |  | 1.72E-13 | 0.00249 | 0.00013 | 54.313 |
| rs855866 | G | A | -0.0154 | 0.80915 |  | 3.17E-08 | 0.00278 | 7.3E-05 | 30.5996 |
| rs863678 | T | G | 0.02806 | 0.64384 |  | 2.49E-33 | 0.00233 | 0.00036 | 144.74 |
| rs9297867 | T | G | -0.0183 | 0.15586 |  | 1.16E-09 | 0.003 | 8.8E-05 | 37.0429 |
| rs9361669 | T | G | -0.0169 | 0.17353 |  | 5.02E-09 | 0.00288 | 8.1E-05 | 34.1824 |
| rs9361858 | G | T | 0.02973 | 0.41797 |  | 9.17E-41 | 0.00222 | 0.00043 | 178.779 |
| rs9409710 | G | A | 0.01553 | 0.3119 |  | 5.10E-11 | 0.00236 | 0.0001 | 43.1437 |
| rs9444543 | A | G | -0.0122 | 0.5358 |  | 3.69E-08 | 0.00222 | 7.4E-05 | 30.3089 |
| rs9747093 | A | G | -0.0128 | 0.45009 |  | 5.25E-09 | 0.00219 | 8.1E-05 | 34.0998 |
| rs9905543 | C | T | 0.03636 | 0.23677 |  | 8.37E-46 | 0.00256 | 0.00048 | 201.877 |
| rs9930127 | G | A | 0.0157 | 0.52521 |  | 6.97E-13 | 0.00219 | 0.00012 | 51.5577 |
