# Supplementary material for: Mendelian randomization study supports the causal association between serum cystatin C and risk of diabetic nephropathy
Source: Front Endocrinol (Lausanne). 2022 Nov 17;13:1043174. doi: 10.3389/fendo.2022.1043174 (PMC9724588; doi:10.3389/fendo.2022.1043174)
Supplement: Supplementary file 2 [file Table_2.docx]

**Supplementary Table 2**: Instrumental variables of total bilirubin. SNP, the rsID of genetic variants; A1, the effect allele; A2, the other allele; Beta, the effect size of A1 on the exposure; Se, the standard error of beta; Proxy, the proxy SNP in the outcome; P, the p-value of beta; R2, the proportion of variance explained by each SNP; F, the F statistic

| SNP | A1 | A2 | Beta | EAF | | Proxy | P | Se | R2 | F |
| --- | --- | --- | --- | --- | --- | --- | --- | --- | --- | --- |
| rs10003923 | C | T | 0.01444 | 0.41903 | |  | 6.91E-10 | 0.00234 | 0.0001 | 38.0475 |
| rs10478063 | T | G | -0.0136 | 0.3244 |  | | 3.68E-08 | 0.00247 | 8.10E-05 | 30.3137 |
| rs1047891 | A | C | 0.02296 | 0.31574 | |  | 1.65E-20 | 0.00247 | 0.00023 | 86.1741 |
| rs10495928 | G | A | -0.0138 | 0.33575 | |  | 1.47E-08 | 0.00243 | 8.48E-05 | 32.0902 |
| rs10793128 | A | G | 0.035 | 0.09233 | |  | 1.71E-18 | 0.00399 | 0.00021 | 77.0107 |
| rs10841556 | G | A | 0.01382 | 0.35896 | |  | 9.15E-09 | 0.0024 | 8.79E-05 | 33.0173 |
| rs10876376 | A | G | 0.01443 | 0.5479 | |  | 5.11E-10 | 0.00232 | 0.0001 | 38.6376 |
| rs11045932 | T | C | -0.0491 | 0.29405 | |  | 4.03E-83 | 0.00254 | 0.001 | 373.257 |
| rs111459884 | A | C | 0.28406 | 0.01511 | |  | 1.64E-172 | 0.01014 | 0.0024 | 784.928 |
| rs112715011 | G | A | 0.09427 | 0.02197 | |  | 2.96E-32 | 0.00797 | 0.00038 | 139.821 |
| rs1131114 | C | T | -0.0191 | 0.26494 | |  | 2.56E-13 | 0.00261 | 0.00014 | 53.5266 |
| rs115035012 | T | C | 0.12721 | 0.01992 | |  | 2.58E-45 | 0.009 | 0.00063 | 199.623 |
| rs11519274 | T | C | 0.17122 | 0.16061 | | rs199795230 | 1.00E-200 | 0.00312 | 0.0079 | 3011.04 |
| rs115700531 | C | T | -0.1207 | 0.01656 | |  | 6.99E-41 | 0.00902 | 0.00047 | 179.315 |
| rs115871533 | G | A | -0.1297 | 0.01037 | |  | 1.27E-28 | 0.01169 | 0.00035 | 123.222 |
| rs11917973 | T | C | 0.01336 | 0.46343 | |  | 8.14E-09 | 0.00232 | 8.88E-05 | 33.2446 |
| rs12651414 | A | C | 0.01487 | 0.23785 | |  | 3.76E-08 | 0.0027 | 8.01E-05 | 30.2718 |
| rs1292042 | G | A | -0.0247 | 0.19815 | |  | 9.82E-18 | 0.00288 | 0.00019 | 73.5561 |
| rs12938360 | C | T | 0.01798 | 0.19197 | | rs9898892 | 1.14E-09 | 0.00295 | 0.0001 | 37.0693 |
| rs13092376 | C | A | 0.01372 | 0.41903 | |  | 4.43E-09 | 0.00234 | 9.16E-05 | 34.4265 |
| rs13171367 | G | A | -0.0193 | 0.19125 | |  | 4.53E-11 | 0.00293 | 0.00012 | 43.3747 |
| rs13421820 | C | T | 0.23689 | 0.00935 | |  | 3.50E-84 | 0.01218 | 0.00104 | 378.143 |
| rs142458770 | G | A | -0.2618 | 0.01511 | |  | 1.96E-150 | 0.01002 | 0.00204 | 683.08 |
| rs1490384 | T | C | -0.0164 | 0.49816 | |  | 9.63E-13 | 0.0023 | 0.00013 | 50.9202 |
| rs150266178 | T | C | 0.19989 | 0.03094 | |  | 2.98E-194 | 0.00672 | 0.0024 | 885.166 |
| rs150754448 | A | G | 0.01794 | 0.15553 | |  | 1.63E-08 | 0.00318 | 8.46E-05 | 31.8974 |
| rs157594 | G | T | 0.01819 | 0.61588 | |  | 1.05E-13 | 0.00245 | 0.00016 | 55.2856 |
| rs1661052 | A | G | -0.0517 | 0.90885 | |  | 3.07E-38 | 0.004 | 0.00044 | 167.214 |
| rs17316633 | A | G | 0.01656 | 0.26445 | |  | 2.10E-10 | 0.00261 | 0.00011 | 40.3699 |
| rs174574 | C | A | 0.02142 | 0.647 | |  | 6.15E-19 | 0.00241 | 0.00021 | 79.0345 |
| rs17476364 | C | T | 0.08285 | 0.10984 | |  | 2.27E-112 | 0.00368 | 0.00134 | 507.827 |
| rs17567190 | T | C | -0.0191 | 0.16525 | |  | 9.02E-10 | 0.00312 | 0.0001 | 37.526 |
| rs1762486 | A | G | 0.01722 | 0.65639 | |  | 2.24E-12 | 0.00245 | 0.00013 | 49.2635 |
| rs17864702 | T | C | -0.2369 | 0.02156 | |  | 3.66E-177 | 0.00834 | 0.00237 | 806.358 |
| rs1800562 | A | G | 0.06285 | 0.07883 | |  | 5.50E-49 | 0.00427 | 0.00057 | 216.472 |
| rs1800759 | G | T | -0.0163 | 0.6139 | |  | 5.83E-12 | 0.00237 | 0.00013 | 47.3919 |
| rs1800961 | T | C | 0.06237 | 0.03143 | |  | 2.88E-21 | 0.00659 | 0.00024 | 89.6385 |
| rs181207 | T | C | -0.0199 | 0.3362 | |  | 3.72E-16 | 0.00244 | 0.00018 | 66.3921 |
| rs1874121 | T | C | 0.02099 | 0.33965 | |  | 6.31E-18 | 0.00243 | 0.0002 | 74.4307 |
| rs1896995 | T | C | -0.03 | 0.485 | |  | 1.85E-38 | 0.00231 | 0.00045 | 168.222 |
| rs1992769 | A | C | -0.013 | 0.5051 | |  | 1.94E-08 | 0.00231 | 8.41E-05 | 31.5536 |
| rs2035403 | G | A | -0.0174 | 0.39977 | |  | 1.25E-13 | 0.00235 | 0.00015 | 54.9371 |
| rs2053799 | G | A | 0.01445 | 0.57547 | |  | 5.82E-10 | 0.00233 | 0.0001 | 38.3807 |
| rs2068888 | A | G | 0.03082 | 0.44882 | |  | 1.25E-40 | 0.00231 | 0.00047 | 178.159 |
| rs2245095 | T | C | -0.043 | 0.08806 | |  | 3.16E-26 | 0.00405 | 0.0003 | 112.263 |
| rs2267846 | A | G | 0.01636 | 0.27281 | |  | 3.40E-10 | 0.00261 | 0.00011 | 39.434 |
| rs2294915 | T | C | 0.01496 | 0.23109 | |  | 4.12E-08 | 0.00273 | 7.95E-05 | 30.0931 |
| rs2519093 | T | C | -0.0211 | 0.18402 | |  | 1.28E-12 | 0.00297 | 0.00013 | 50.3582 |
| rs2522051 | C | T | -0.0154 | 0.45369 | |  | 2.90E-11 | 0.00231 | 0.00012 | 44.2445 |
| rs2535613 | G | A | -0.0155 | 0.6299 | |  | 4.17E-10 | 0.00249 | 0.00011 | 39.0368 |
| rs2657878 | T | C | 0.01754 | 0.17899 | |  | 5.73E-09 | 0.00301 | 9.04E-05 | 33.9268 |
| rs2792759 | T | C | -0.0193 | 0.72584 | |  | 7.11E-14 | 0.00258 | 0.00015 | 56.0437 |
| rs28571093 | G | A | -0.2044 | 0.03117 | |  | 1.00E-200 | 0.00662 | 0.00252 | 952.494 |
| rs2978457 | C | T | -0.0133 | 0.48175 | |  | 6.98E-09 | 0.0023 | 8.88E-05 | 33.5453 |
| rs3117106 | C | T | -0.0258 | 0.16181 | |  | 1.24E-16 | 0.00312 | 0.00018 | 68.5435 |
| rs33963055 | T | C | 0.01848 | 0.17089 | |  | 1.50E-09 | 0.00306 | 9.68E-05 | 36.5429 |
| rs339969 | A | C | -0.0244 | 0.61486 | |  | 4.66E-25 | 0.00236 | 0.00028 | 106.93 |
| rs34979631 | T | C | 0.01548 | 0.22557 | |  | 2.00E-08 | 0.00276 | 8.37E-05 | 31.494 |
| rs3768321 | T | G | -0.0197 | 0.19677 | |  | 9.72E-12 | 0.0029 | 0.00012 | 46.3858 |
| rs4129887 | C | T | -0.0128 | 0.5633 | |  | 3.37E-08 | 0.00231 | 8.03E-05 | 30.4829 |
| rs4243177 | G | A | 0.01484 | 0.62495 | |  | 3.63E-10 | 0.00237 | 0.0001 | 39.3078 |
| rs4410790 | C | T | -0.0301 | 0.63423 | |  | 2.12E-36 | 0.00239 | 0.00042 | 158.793 |
| rs45521138 | T | C | -0.3105 | 0.01062 | |  | 2.54E-138 | 0.0124 | 0.00203 | 627.341 |
| rs4671605 | T | C | 0.02043 | 0.73446 | |  | 6.23E-15 | 0.00262 | 0.00016 | 60.8374 |
| rs4737010 | A | G | -0.03 | 0.22802 | |  | 7.87E-28 | 0.00274 | 0.00032 | 119.587 |
| rs4790908 | G | T | -0.0184 | 0.19444 | |  | 2.75E-10 | 0.00291 | 0.00011 | 39.8508 |
| rs4808579 | C | T | -0.0184 | 0.39175 | |  | 7.06E-15 | 0.00236 | 0.00016 | 60.5906 |
| rs4925546 | G | A | -0.0156 | 0.63035 | |  | 5.58E-11 | 0.00238 | 0.00011 | 42.9636 |
| rs5023357 | G | T | -0.0241 | 0.10882 | | rs4340803 | 6.66E-11 | 0.0037 | 0.00011 | 42.6198 |
| rs55696093 | G | A | 0.02352 | 0.21972 | |  | 2.64E-17 | 0.00278 | 0.00019 | 71.6056 |
| rs56094562 | C | A | 0.02555 | 0.34479 | |  | 5.39E-26 | 0.00242 | 0.00029 | 111.202 |
| rs58542926 | T | C | 0.03961 | 0.07548 | |  | 9.52E-20 | 0.00436 | 0.00022 | 82.7164 |
| rs6129760 | G | A | 0.01747 | 0.32286 | |  | 1.26E-12 | 0.00246 | 0.00013 | 50.3938 |
| rs61750929 | T | C | 0.04855 | 0.05506 | |  | 7.71E-22 | 0.00505 | 0.00025 | 92.2465 |
| rs62020698 | T | C | -0.0229 | 0.09171 | |  | 8.48E-09 | 0.00398 | 8.75E-05 | 33.1641 |
| rs6734238 | G | A | -0.0218 | 0.40203 | |  | 1.53E-20 | 0.00234 | 0.00023 | 86.3333 |
| rs676388 | C | T | 0.01906 | 0.53588 | |  | 1.77E-16 | 0.00231 | 0.00018 | 67.846 |
| rs6802898 | T | C | -0.0203 | 0.12224 | |  | 6.81E-09 | 0.0035 | 8.84E-05 | 33.5895 |
| rs687339 | T | C | -0.0488 | 0.77192 | |  | 9.74E-71 | 0.00274 | 0.00084 | 316.358 |
| rs7139079 | A | G | 0.01424 | 0.5949 | |  | 1.31E-09 | 0.00235 | 9.77E-05 | 36.7998 |
| rs7214175 | T | C | -0.0169 | 0.17075 | |  | 3.56E-08 | 0.00306 | 8.07E-05 | 30.3792 |
| rs7222046 | G | A | 0.01674 | 0.4314 | |  | 7.78E-13 | 0.00234 | 0.00014 | 51.3416 |
| rs72679504 | A | C | 0.0421 | 0.04023 | |  | 8.02E-13 | 0.00588 | 0.00014 | 51.2825 |
| rs72835688 | T | C | -0.0289 | 0.05943 | |  | 8.97E-09 | 0.00502 | 9.32E-05 | 33.0538 |
| rs730551 | T | C | 0.01424 | 0.48002 | |  | 7.34E-10 | 0.00231 | 0.0001 | 37.9272 |
| rs7526446 | A | C | 0.01991 | 0.63709 | |  | 1.82E-16 | 0.00242 | 0.00018 | 67.7988 |
| rs7667722 | A | G | -0.0146 | 0.50439 | |  | 2.34E-10 | 0.0023 | 0.00011 | 40.162 |
| rs76895963 | G | T | 0.05351 | 0.02108 | |  | 1.33E-09 | 0.00882 | 0.00012 | 36.7745 |
| rs7894137 | T | C | -0.0243 | 0.15371 | |  | 2.63E-14 | 0.00318 | 0.00015 | 57.9993 |
| rs79170239 | T | C | 0.03334 | 0.18799 | |  | 7.26E-30 | 0.00294 | 0.00034 | 128.892 |
| rs7947951 | G | A | -0.0223 | 0.69118 | |  | 3.41E-19 | 0.00249 | 0.00021 | 80.1933 |
| rs79485702 | G | A | 0.01476 | 0.28916 | | rs1525868 | 1.43E-08 | 0.0026 | 8.95E-05 | 32.1494 |
| rs8041523 | T | C | 0.01301 | 0.42586 | |  | 2.24E-08 | 0.00233 | 8.27E-05 | 31.2765 |
| rs855791 | G | A | 0.02869 | 0.56166 | |  | 5.48E-35 | 0.00232 | 0.00041 | 152.318 |
| rs857720 | C | T | -0.0322 | 0.26657 | |  | 3.29E-35 | 0.0026 | 0.0004 | 153.335 |
| rs873642 | T | C | -0.0194 | 0.12251 | |  | 3.04E-08 | 0.00351 | 8.12E-05 | 30.6804 |
| rs887829 | T | C | 0.74665 | 0.31492 | |  | 1.00E-200 | 0.00212 | 0.24055 | 124017 |
| rs9438901 | A | G | 0.01806 | 0.85781 | |  | 4.06E-08 | 0.00329 | 7.96E-05 | 30.1254 |
| rs9826148 | T | C | -0.024 | 0.10871 | |  | 9.91E-11 | 0.00371 | 0.00011 | 41.8429 |
