# Supplementary material for: Mendelian randomization study supports the causal association between serum cystatin C and risk of diabetic nephropathy
Source: Front Endocrinol (Lausanne). 2022 Nov 17;13:1043174. doi: 10.3389/fendo.2022.1043174 (PMC9724588; doi:10.3389/fendo.2022.1043174)
Supplement: Supplementary file 3 [file Table_3.docx]

**Supplementary Table 3**: Instrumental variables of urate. SNP, the rsID of genetic variants; A1, the effect allele; A2, the other allele; Beta, the effect size of A1 on the exposure; Se, the standard error of beta; Proxy, the proxy SNP in the outcome; P, the p-value of beta; R2, the proportion of variance explained by each SNP; F, the F statistic

| SNP | A1 | A2 | Beta | EAF | Proxy | P | Se | R2 | F |
| --- | --- | --- | --- | --- | --- | --- | --- | --- | --- |
| rs10064782 | G | A | 0.01535 | 0.25305 |  | 6.28E-11 | 0.00235 | 8.90E-05 | 42.7363 |
| rs10160397 | C | T | 0.01144 | 0.39265 |  | 3.90E-08 | 0.00208 | 6.24E-05 | 30.2025 |
| rs10164318 | G | T | -0.0197 | 0.67313 |  | 1.09E-19 | 0.00217 | 0.00017 | 82.4579 |
| rs10193587 | C | T | -0.0135 | 0.23066 |  | 3.09E-08 | 0.00244 | 6.45E-05 | 30.6522 |
| rs10196697 | A | G | -0.0118 | 0.39092 |  | 1.73E-08 | 0.00209 | 6.58E-05 | 31.776 |
| rs10210970 | T | C | 0.01768 | 0.12894 |  | 5.45E-09 | 0.00303 | 7.02E-05 | 34.0233 |
| rs10224210 | C | T | 0.02669 | 0.28247 |  | 3.27E-32 | 0.00226 | 0.00029 | 139.625 |
| rs10405423 | A | C | 0.02302 | 0.65781 |  | 2.10E-26 | 0.00216 | 0.00024 | 113.08 |
| rs1047891 | A | C | -0.0276 | 0.31573 |  | 1.82E-36 | 0.00219 | 0.00033 | 159.105 |
| rs10754894 | A | G | -0.0215 | 0.31406 |  | 7.44E-23 | 0.00219 | 0.0002 | 96.8783 |
| rs10782230 | A | G | 0.01481 | 0.48093 |  | 3.35E-13 | 0.00203 | 0.00011 | 52.9953 |
| rs10797999 | T | C | -0.013 | 0.41098 |  | 2.97E-10 | 0.00207 | 8.24E-05 | 39.6968 |
| rs10846156 | G | T | -0.0165 | 0.20201 | rs10846157 | 8.18E-11 | 0.00254 | 8.76E-05 | 42.2185 |
| rs10886117 | A | G | 0.02019 | 0.16706 |  | 1.39E-13 | 0.00273 | 0.00011 | 54.7192 |
| rs10935686 | C | T | -0.0126 | 0.56061 |  | 6.75E-10 | 0.00205 | 7.85E-05 | 38.0955 |
| rs11072567 | G | A | 0.0307 | 0.51215 |  | 1.52E-51 | 0.00203 | 0.00047 | 228.222 |
| rs11108219 | C | T | 0.01709 | 0.22117 |  | 3.11E-12 | 0.00245 | 0.0001 | 48.6231 |
| rs11128603 | G | A | -0.0215 | 0.12134 |  | 4.84E-12 | 0.00311 | 9.84E-05 | 47.7527 |
| rs111295548 | G | A | -0.0314 | 0.034229 |  | 2.02E-08 | 0.0056 | 6.52E-05 | 31.4723 |
| rs111346856 | A | G | 0.01397 | 0.27703 |  | 7.94E-10 | 0.00227 | 7.81E-05 | 37.777 |
| rs11163481 | T | G | 0.01334 | 0.67037 |  | 7.43E-10 | 0.00217 | 7.87E-05 | 37.9096 |
| rs11202328 | T | C | -0.0255 | 0.13132 |  | 2.39E-17 | 0.00301 | 0.00015 | 71.8004 |
| rs11218783 | A | G | 0.01141 | 0.43061 |  | 2.94E-08 | 0.00206 | 6.39E-05 | 30.745 |
| rs11564722 | T | C | -0.0261 | 0.23803 |  | 3.74E-27 | 0.00242 | 0.00025 | 116.492 |
| rs11600628 | T | C | -0.0312 | 0.17498 |  | 1.49E-31 | 0.00267 | 0.00028 | 136.604 |
| rs116402366 | A | G | 0.05547 | 0.025027 |  | 8.22E-17 | 0.00666 | 0.00015 | 69.3644 |
| rs11693363 | C | A | -0.0305 | 0.13087 |  | 3.89E-24 | 0.00301 | 0.00021 | 102.72 |
| rs1171614 | C | T | 0.05629 | 0.76883 |  | 2.14E-120 | 0.00241 | 0.00113 | 544.773 |
| rs11748431 | A | G | -0.0128 | 0.2619 |  | 3.95E-08 | 0.00232 | 6.30E-05 | 30.1736 |
| rs11835818 | C | T | -0.0186 | 0.47895 |  | 5.83E-20 | 0.00204 | 0.00017 | 83.6839 |
| rs11854957 | T | C | -0.0144 | 0.21878 |  | 4.86E-09 | 0.00246 | 7.06E-05 | 34.2467 |
| rs1186705 | C | A | -0.0136 | 0.3475 |  | 2.00E-10 | 0.00214 | 8.42E-05 | 40.4672 |
| rs12133907 | A | C | 0.01917 | 0.6023 |  | 2.36E-20 | 0.00207 | 0.00018 | 85.4744 |
| rs12144369 | T | C | -0.012 | 0.35829 |  | 2.58E-08 | 0.00216 | 6.67E-05 | 31.0007 |
| rs1229984 | C | T | -0.06 | 0.977726 |  | 2.84E-18 | 0.00689 | 0.00016 | 76.0039 |
| rs12316443 | A | C | -0.0151 | 0.30511 |  | 1.03E-11 | 0.00222 | 9.69E-05 | 46.2664 |
| rs12363578 | T | C | -0.0556 | 0.4301 |  | 6.85E-159 | 0.00207 | 0.00152 | 722.092 |
| rs12371604 | C | T | -0.0143 | 0.21843 |  | 5.71E-09 | 0.00246 | 7.00E-05 | 33.9321 |
| rs12443147 | T | C | -0.0148 | 0.2135 |  | 3.06E-09 | 0.00249 | 7.34E-05 | 35.1446 |
| rs12554192 | G | A | 0.01446 | 0.26969 |  | 2.69E-10 | 0.00229 | 8.23E-05 | 39.8866 |
| rs12576996 | G | T | 0.03751 | 0.24609 |  | 2.74E-56 | 0.00237 | 0.00052 | 249.984 |
| rs12602520 | C | A | 0.02027 | 0.086525 |  | 1.99E-08 | 0.00361 | 6.50E-05 | 31.5035 |
| rs1260326 | C | T | -0.049 | 0.60689 |  | 5.45E-123 | 0.00208 | 0.00115 | 556.708 |
| rs12697963 | T | C | 0.0145 | 0.72073 |  | 1.91E-10 | 0.00228 | 8.46E-05 | 40.565 |
| rs12708477 | C | A | -0.014 | 0.77225 |  | 7.06E-09 | 0.00243 | 6.94E-05 | 33.5183 |
| rs12882763 | T | C | -0.0119 | 0.66188 | rs2092914 | 3.33E-08 | 0.00215 | 6.32E-05 | 30.5054 |
| rs13107325 | T | C | -0.031 | 0.074844 |  | 1.00E-15 | 0.00387 | 0.00013 | 64.4368 |
| rs13130003 | G | A | -0.038 | 0.72499 |  | 3.01E-62 | 0.00228 | 0.00058 | 277.335 |
| rs1317983 | C | T | 0.03169 | 0.69388 |  | 5.57E-47 | 0.0022 | 0.00043 | 207.272 |
| rs13198778 | T | C | -0.0116 | 0.36381 |  | 4.39E-08 | 0.00211 | 6.19E-05 | 29.9693 |
| rs13240994 | C | T | -0.0349 | 0.19956 | rs13226650 | 5.09E-43 | 0.00254 | 0.00039 | 189.115 |
| rs13411042 | A | C | 0.01667 | 0.48462 |  | 2.63E-16 | 0.00204 | 0.00014 | 67.0635 |
| rs138660816 | A | G | 0.05406 | 0.01232 |  | 2.88E-08 | 0.00974 | 7.11E-05 | 30.7868 |
| rs140254647 | C | T | -0.0158 | 0.21545 |  | 2.09E-10 | 0.00248 | 8.40E-05 | 40.384 |
| rs141471965 | T | C | 0.17118 | 0.1145 |  | 1.00E-200 | 0.00318 | 0.00594 | 2903.71 |
| rs1440411 | T | C | -0.0153 | 0.57316 |  | 1.06E-13 | 0.00206 | 0.00012 | 55.2514 |
| rs1472866 | A | G | -0.0117 | 0.5016 |  | 1.20E-08 | 0.00204 | 6.79E-05 | 32.4951 |
| rs148179165 | A | C | 0.01624 | 0.30611 |  | 2.59E-13 | 0.00222 | 0.00011 | 53.5047 |
| rs148185902 | A | G | 0.06347 | 0.013808 |  | 5.43E-12 | 0.00921 | 0.00011 | 47.5305 |
| rs1541939 | G | T | 0.01422 | 0.25549 |  | 1.18E-09 | 0.00234 | 7.69E-05 | 37.0026 |
| rs16890979 | T | C | -0.3122 | 0.22331 |  | 1.00E-200 | 0.00232 | 0.03381 | 18185.5 |
| rs16951334 | G | A | 0.02156 | 0.10671 |  | 6.01E-11 | 0.00329 | 8.86E-05 | 42.8193 |
| rs17024258 | T | C | 0.03598 | 0.02586 |  | 2.12E-08 | 0.00642 | 6.52E-05 | 31.379 |
| rs17050272 | A | G | 0.02537 | 0.40966 |  | 1.22E-34 | 0.00207 | 0.00031 | 150.729 |
| rs1705614 | T | C | 0.02217 | 0.72452 |  | 2.12E-22 | 0.00228 | 0.0002 | 94.7982 |
| rs17624477 | C | T | 0.02721 | 0.063891 |  | 5.73E-11 | 0.00415 | 8.86E-05 | 42.9151 |
| rs17817497 | C | T | 0.01779 | 0.39284 |  | 1.34E-17 | 0.00208 | 0.00015 | 72.9447 |
| rs1800961 | T | C | -0.0504 | 0.031412 |  | 5.23E-18 | 0.00583 | 0.00015 | 74.801 |
| rs181673 | C | A | 0.01849 | 0.53613 |  | 1.06E-19 | 0.00204 | 0.00017 | 82.5115 |
| rs193220 | T | C | -0.0158 | 0.31734 |  | 5.20E-13 | 0.00219 | 0.00011 | 52.1293 |
| rs1949651 | T | C | 0.01278 | 0.47115 |  | 4.23E-10 | 0.00205 | 8.13E-05 | 39.009 |
| rs195485 | T | C | -0.013 | 0.38469 |  | 5.35E-10 | 0.00209 | 8.01E-05 | 38.5444 |
| rs1965132 | A | C | 0.01552 | 0.49442 |  | 3.75E-14 | 0.00205 | 0.00012 | 57.3008 |
| rs1991371 | A | G | 0.02599 | 0.11941 |  | 1.12E-16 | 0.00313 | 0.00014 | 68.7513 |
| rs2108093 | G | A | 0.01563 | 0.79835 |  | 7.02E-10 | 0.00253 | 7.86E-05 | 38.0183 |
| rs2195525 | T | C | -0.015 | 0.52068 |  | 3.21E-13 | 0.00205 | 0.00011 | 53.084 |
| rs219787 | T | C | -0.0225 | 0.2628 |  | 1.66E-22 | 0.00231 | 0.0002 | 95.2853 |
| rs2229357 | A | G | -0.048 | 0.24372 |  | 8.53E-92 | 0.00236 | 0.00085 | 413.137 |
| rs2240390 | C | T | -0.0144 | 0.3936 |  | 6.02E-12 | 0.00209 | 9.88E-05 | 47.3298 |
| rs2244552 | G | A | 0.0327 | 0.42698 |  | 4.83E-57 | 0.00205 | 0.00052 | 253.439 |
| rs2258043 | C | T | -0.0131 | 0.49006 |  | 1.31E-10 | 0.00203 | 8.54E-05 | 41.3017 |
| rs2269863 | T | C | 0.01364 | 0.59176 |  | 4.07E-11 | 0.00207 | 8.99E-05 | 43.5817 |
| rs2345962 | G | A | 0.01517 | 0.49877 |  | 9.88E-14 | 0.00204 | 0.00012 | 55.3961 |
| rs2394685 | G | A | -0.0151 | 0.51303 |  | 1.30E-13 | 0.00203 | 0.00011 | 54.8632 |
| rs2436958 | C | T | -0.0213 | 0.85661 |  | 2.14E-13 | 0.0029 | 0.00011 | 53.8781 |
| rs2437817 | A | C | -0.0152 | 0.33016 |  | 2.54E-12 | 0.00218 | 0.0001 | 49.0225 |
| rs2439823 | G | A | 0.01167 | 0.54673 |  | 1.19E-08 | 0.00205 | 6.75E-05 | 32.5001 |
| rs2480714 | T | G | 0.01372 | 0.69836 |  | 9.82E-10 | 0.00224 | 7.93E-05 | 37.3666 |
| rs2540034 | T | C | 0.01172 | 0.57106 |  | 1.98E-08 | 0.00209 | 6.73E-05 | 31.5192 |
| rs255751 | T | C | 0.01923 | 0.69907 |  | 5.05E-18 | 0.00222 | 0.00016 | 74.8633 |
| rs2636590 | A | G | 0.01158 | 0.61313 |  | 2.97E-08 | 0.00209 | 6.36E-05 | 30.7308 |
| rs2688 | T | G | -0.0115 | 0.60656 |  | 3.91E-08 | 0.00209 | 6.27E-05 | 30.1917 |
| rs2695580 | A | G | 0.01272 | 0.41605 |  | 1.37E-09 | 0.0021 | 7.86E-05 | 36.718 |
| rs2788144 | G | A | 0.03946 | 0.03673 |  | 3.39E-13 | 0.00542 | 0.00011 | 52.9741 |
| rs2817188 | G | A | 0.06698 | 0.56825 |  | 1.00E-200 | 0.00205 | 0.0022 | 1068.3 |
| rs2823139 | A | G | 0.01573 | 0.33841 |  | 3.05E-13 | 0.00216 | 0.00011 | 53.1736 |
| rs2834317 | A | G | 0.01912 | 0.15374 |  | 1.58E-11 | 0.00284 | 9.51E-05 | 45.434 |
| rs28371924 | T | G | -0.0125 | 0.498 |  | 9.59E-10 | 0.00205 | 7.85E-05 | 37.405 |
| rs28384289 | T | C | 0.02853 | 0.14729 |  | 2.16E-23 | 0.00286 | 0.0002 | 99.3234 |
| rs28517717 | T | C | -0.023 | 0.27454 |  | 1.66E-23 | 0.0023 | 0.00021 | 99.8433 |
| rs2941484 | T | C | 0.03185 | 0.44541 |  | 4.05E-54 | 0.00206 | 0.0005 | 240.034 |
| rs2943654 | T | C | 0.0132 | 0.64567 |  | 5.20E-10 | 0.00212 | 7.97E-05 | 38.604 |
| rs2973444 | C | T | -0.0252 | 0.085758 |  | 4.88E-12 | 0.00364 | 9.93E-05 | 47.7359 |
| rs3184504 | C | T | -0.0288 | 0.51798 |  | 1.40E-45 | 0.00203 | 0.00041 | 200.842 |
| rs33938520 | T | C | -0.0135 | 0.25276 |  | 9.79E-09 | 0.00235 | 6.83E-05 | 32.8832 |
| rs345748 | G | A | 0.01509 | 0.75257 |  | 1.46E-10 | 0.00235 | 8.48E-05 | 41.0878 |
| rs34811474 | A | G | -0.014 | 0.23213 |  | 6.04E-09 | 0.00241 | 6.98E-05 | 33.8232 |
| rs35016056 | G | T | 0.0135 | 0.54977 |  | 4.19E-11 | 0.00205 | 9.02E-05 | 43.5262 |
| rs35184622 | G | A | 0.01397 | 0.32728 |  | 5.20E-10 | 0.00225 | 8.59E-05 | 38.5989 |
| rs354532 | T | C | 0.01214 | 0.53859 |  | 3.01E-09 | 0.00205 | 7.32E-05 | 35.1812 |
| rs358226 | G | A | 0.01785 | 0.85401 |  | 5.43E-10 | 0.00288 | 7.94E-05 | 38.5195 |
| rs36071802 | C | T | 0.02548 | 0.41557 | rs7007761 | 5.98E-34 | 0.0021 | 0.00032 | 147.576 |
| rs3746574 | C | T | -0.0149 | 0.51529 |  | 5.43E-13 | 0.00207 | 0.00011 | 52.0479 |
| rs3824359 | C | T | 0.01921 | 0.14193 |  | 4.15E-11 | 0.00291 | 8.99E-05 | 43.5468 |
| rs3925584 | C | T | -0.0254 | 0.45463 |  | 8.49E-36 | 0.00204 | 0.00032 | 156.026 |
| rs40270 | C | A | 0.02096 | 0.77305 |  | 7.33E-17 | 0.00251 | 0.00015 | 69.5927 |
| rs41264869 | T | C | 0.01621 | 0.17848 |  | 1.02E-09 | 0.00266 | 7.71E-05 | 37.2894 |
| rs41290716 | A | C | 0.03563 | 0.070006 |  | 3.43E-19 | 0.00398 | 0.00017 | 80.1837 |
| rs4140872 | T | C | -0.0282 | 0.75583 |  | 1.04E-32 | 0.00237 | 0.00029 | 141.891 |
| rs423144 | T | G | -0.0362 | 0.42619 |  | 1.73E-69 | 0.00205 | 0.00064 | 310.594 |
| rs4293567 | A | G | 0.02085 | 0.31451 |  | 1.42E-21 | 0.00219 | 0.00019 | 91.0387 |
| rs429358 | C | T | -0.0177 | 0.15624 |  | 2.41E-10 | 0.0028 | 8.28E-05 | 40.1073 |
| rs4415952 | C | T | 0.01415 | 0.73315 |  | 7.56E-10 | 0.0023 | 7.84E-05 | 37.8744 |
| rs4441044 | A | G | 0.01166 | 0.64433 |  | 3.87E-08 | 0.00212 | 6.23E-05 | 30.2178 |
| rs4468717 | T | C | -0.0214 | 0.077674 |  | 1.74E-08 | 0.00379 | 6.55E-05 | 31.7649 |
| rs45487598 | A | G | 0.01837 | 0.11689 |  | 7.71E-09 | 0.00318 | 6.97E-05 | 33.3487 |
| rs455213 | C | T | 0.0181 | 0.4578 |  | 7.82E-19 | 0.00204 | 0.00016 | 78.5554 |
| rs4575545 | A | G | -0.0261 | 0.30716 |  | 4.47E-32 | 0.00221 | 0.00029 | 138.994 |
| rs4693210 | G | A | -0.0157 | 0.43773 |  | 1.93E-14 | 0.00205 | 0.00012 | 58.6085 |
| rs4744712 | C | A | 0.01358 | 0.60157 |  | 6.14E-11 | 0.00208 | 8.85E-05 | 42.7784 |
| rs4751640 | C | A | -0.0127 | 0.6938 |  | 1.09E-08 | 0.00222 | 6.86E-05 | 32.6722 |
| rs478425 | T | G | -0.0139 | 0.36954 |  | 3.67E-11 | 0.00211 | 9.05E-05 | 43.7864 |
| rs4962687 | A | G | -0.0163 | 0.68142 |  | 7.81E-14 | 0.00219 | 0.00012 | 55.8611 |
| rs4966019 | T | C | -0.0336 | 0.6405 |  | 1.66E-56 | 0.00212 | 0.00052 | 250.978 |
| rs508205 | A | G | 0.01435 | 0.55818 |  | 2.30E-12 | 0.00205 | 0.0001 | 49.2083 |
| rs538656 | T | G | 0.02055 | 0.23401 |  | 1.08E-17 | 0.0024 | 0.00015 | 73.3735 |
| rs541091 | A | G | -0.013 | 0.47483 |  | 1.54E-10 | 0.00204 | 8.48E-05 | 40.9846 |
| rs541564 | A | G | 0.01446 | 0.35411 |  | 1.31E-11 | 0.00214 | 9.56E-05 | 45.7985 |
| rs56379622 | A | G | -0.0374 | 0.0447 |  | 2.90E-14 | 0.00492 | 0.00012 | 57.8025 |
| rs57158761 | G | A | 0.01311 | 0.43663 |  | 1.69E-10 | 0.00205 | 8.45E-05 | 40.7958 |
| rs580241 | A | G | -0.016 | 0.76343 | rs10791866 | 5.58E-11 | 0.00245 | 9.29E-05 | 42.9672 |
| rs603424 | A | G | 0.01522 | 0.168 |  | 2.15E-08 | 0.00272 | 6.47E-05 | 31.3499 |
| rs60767324 | C | T | -0.0201 | 0.086706 |  | 2.85E-08 | 0.00362 | 6.40E-05 | 30.8066 |
| rs6142206 | A | G | 0.01647 | 0.42115 |  | 1.28E-15 | 0.00206 | 0.00013 | 63.9612 |
| rs62106258 | C | T | -0.0342 | 0.048343 |  | 5.12E-13 | 0.00474 | 0.00011 | 52.1642 |
| rs62294340 | A | G | -0.0178 | 0.38339 |  | 1.72E-17 | 0.00209 | 0.00015 | 72.4472 |
| rs62435145 | T | G | 0.03359 | 0.69192 |  | 1.64E-49 | 0.00227 | 0.00048 | 218.885 |
| rs62580785 | C | T | -0.0146 | 0.17947 | rs6477754 | 4.50E-08 | 0.00267 | 6.26E-05 | 29.9208 |
| rs6499163 | T | G | -0.0157 | 0.17424 |  | 4.81E-09 | 0.00268 | 7.07E-05 | 34.2686 |
| rs676015 | C | T | -0.0145 | 0.6312 |  | 9.76E-12 | 0.00213 | 9.82E-05 | 46.3827 |
| rs6774307 | C | T | 0.02398 | 0.077872 | rs28374787 | 2.96E-10 | 0.00381 | 8.26E-05 | 39.7016 |
| rs686364 | G | A | 0.01835 | 0.2346 |  | 1.88E-14 | 0.0024 | 0.00012 | 58.6613 |
| rs6965823 | A | C | -0.0134 | 0.32213 |  | 7.21E-10 | 0.00217 | 7.81E-05 | 37.9667 |
| rs700750 | A | C | 0.01448 | 0.62887 |  | 5.67E-12 | 0.0021 | 9.79E-05 | 47.4424 |
| rs7093087 | A | G | 0.01776 | 0.17448 | rs12255761 | 3.40E-11 | 0.00268 | 9.09E-05 | 43.9367 |
| rs7154553 | G | A | 0.01737 | 0.19838 |  | 1.04E-11 | 0.00255 | 9.59E-05 | 46.257 |
| rs7179427 | A | G | 0.01604 | 0.72778 |  | 2.22E-12 | 0.00229 | 0.0001 | 49.2822 |
| rs7224610 | A | C | -0.0288 | 0.60224 |  | 5.23E-43 | 0.00209 | 0.0004 | 189.058 |
| rs72681698 | C | T | -0.0717 | 0.011039 |  | 1.64E-13 | 0.00972 | 0.00011 | 54.4003 |
| rs72799820 | T | C | -0.017 | 0.14999 |  | 2.70E-09 | 0.00285 | 7.34E-05 | 35.389 |
| rs72818964 | A | G | 0.01554 | 0.17609 |  | 5.59E-09 | 0.00267 | 7.00E-05 | 33.9727 |
| rs72951456 | T | C | 0.02644 | 0.048935 |  | 3.17E-08 | 0.00478 | 6.50E-05 | 30.6025 |
| rs7302925 | G | A | -0.021 | 0.80212 |  | 1.48E-16 | 0.00255 | 0.00014 | 68.1923 |
| rs738408 | T | C | -0.0193 | 0.21583 |  | 4.83E-15 | 0.00247 | 0.00013 | 61.3371 |
| rs74606487 | G | A | -0.0165 | 0.15062 |  | 1.31E-08 | 0.00291 | 6.98E-05 | 32.3095 |
| rs7461961 | A | G | 0.01228 | 0.54612 |  | 2.57E-09 | 0.00206 | 7.47E-05 | 35.486 |
| rs75588192 | A | G | 0.02317 | 0.13826 |  | 9.96E-15 | 0.00299 | 0.00013 | 59.908 |
| rs75964023 | T | C | -0.1455 | 0.039752 | rs13144899 | 1.41E-170 | 0.00522 | 0.00162 | 776.02 |
| rs76358556 | G | A | -0.0216 | 0.20379 |  | 1.28E-17 | 0.00252 | 0.00015 | 73.0292 |
| rs76895963 | G | T | -0.0557 | 0.021069 |  | 9.76E-13 | 0.0078 | 0.00013 | 50.8947 |
| rs7696556 | C | A | -0.0174 | 0.1395 |  | 3.02E-09 | 0.00294 | 7.28E-05 | 35.1706 |
| rs7736102 | A | G | 0.01467 | 0.56872 |  | 9.15E-13 | 0.00205 | 0.00011 | 51.0274 |
| rs77542162 | G | A | -0.0534 | 0.022979 |  | 3.49E-15 | 0.00679 | 0.00013 | 61.9733 |
| rs7779637 | G | A | -0.0143 | 0.5475 |  | 2.64E-12 | 0.00205 | 0.0001 | 48.9412 |
| rs78177245 | T | C | -0.0353 | 0.027568 |  | 1.16E-08 | 0.00619 | 6.70E-05 | 32.5503 |
| rs784257 | C | T | 0.01488 | 0.81295 |  | 1.34E-08 | 0.00262 | 6.73E-05 | 32.2658 |
| rs79239275 | A | C | 0.02723 | 0.045961 |  | 2.06E-08 | 0.00486 | 6.50E-05 | 31.4424 |
| rs7952403 | G | A | -0.0172 | 0.87396 |  | 2.02E-08 | 0.00306 | 6.51E-05 | 31.479 |
| rs8039645 | A | C | 0.01416 | 0.2265 |  | 1.05E-08 | 0.00247 | 7.02E-05 | 32.7389 |
| rs807624 | T | G | -0.0187 | 0.35776 |  | 9.06E-19 | 0.00212 | 0.00016 | 78.267 |
| rs833805 | G | A | 0.02643 | 0.8848 |  | 2.40E-15 | 0.00334 | 0.00014 | 62.7141 |
| rs836968 | T | C | -0.0147 | 0.27078 |  | 1.56E-10 | 0.0023 | 8.59E-05 | 40.9536 |
| rs854917 | T | C | -0.0128 | 0.73758 |  | 3.60E-08 | 0.00232 | 6.34E-05 | 30.3586 |
| rs871375 | A | G | -0.0181 | 0.65649 |  | 2.33E-17 | 0.00214 | 0.00015 | 71.8521 |
| rs9297949 | C | A | 0.02015 | 0.52978 |  | 4.05E-23 | 0.00203 | 0.0002 | 98.0824 |
| rs9761429 | G | A | -0.0341 | 0.24828 | rs6841523 | 1.28E-45 | 0.0024 | 0.00043 | 201.043 |
| rs9807214 | A | G | 0.01282 | 0.30265 |  | 7.07E-09 | 0.00221 | 6.94E-05 | 33.5183 |
| rs9932625 | A | G | 0.01939 | 0.22886 |  | 1.25E-15 | 0.00242 | 0.00013 | 63.9921 |
