# Supplementary material for: Mendelian randomization study supports the causal association between serum cystatin C and risk of diabetic nephropathy
Source: Front Endocrinol (Lausanne). 2022 Nov 17;13:1043174. doi: 10.3389/fendo.2022.1043174 (PMC9724588; doi:10.3389/fendo.2022.1043174)
Supplement: Supplementary file 4 [file Table_4.docx]

**Supplementary Table 4**: Instrumental variables of creatinine. SNP, the rsID of genetic variants; A1, the effect allele; A2, the other allele; Beta, the effect size of A1 on the exposure; Se, the standard error of beta; Proxy, the proxy SNP in the outcome; P, the p-value of beta; R2, the proportion of variance explained by each SNP; F, the F statistic

| SNP | A1 | A2 | Beta | EAF | Proxy | P | Se | R2 | F |
| --- | --- | --- | --- | --- | --- | --- | --- | --- | --- |
| rs10062079 | A | G | 0.03999 | 0.42301 |  | 7.83E-92 | 0.00197 | 0.00078 | 413.35 |
| rs10420976 | G | T | 0.01094 | 0.57387 |  | 2.46E-08 | 0.00196 | 5.85E-05 | 31.0868 |
| rs1047891 | A | C | 0.0428 | 0.31575 |  | 9.57E-94 | 0.00208 | 0.00079 | 422.132 |
| rs10492436 | T | C | -0.0136 | 0.38427 |  | 7.03E-12 | 0.00199 | 8.81E-05 | 47.0245 |
| rs10493872 | T | G | 0.01359 | 0.31365 |  | 1.09E-10 | 0.00211 | 7.95E-05 | 41.6544 |
| rs1053764 | G | A | -0.0463 | 0.028289 |  | 3.65E-13 | 0.00636 | 0.00012 | 52.8288 |
| rs1076780 | G | A | -0.0134 | 0.71571 |  | 4.84E-10 | 0.00216 | 7.34E-05 | 38.7437 |
| rs10769256 | T | C | 0.01274 | 0.40291 |  | 1.29E-10 | 0.00198 | 7.81E-05 | 41.3256 |
| rs10790456 | A | G | -0.0164 | 0.79571 |  | 1.04E-11 | 0.00242 | 8.79E-05 | 46.2544 |
| rs10821905 | A | G | -0.031 | 0.17908 |  | 3.89E-34 | 0.00254 | 0.00028 | 148.424 |
| rs10881997 | C | T | -0.0148 | 0.37752 | rs10881998 | 1.44E-13 | 0.002 | 0.0001 | 54.6584 |
| rs10898707 | C | T | -0.0117 | 0.4008 |  | 3.67E-09 | 0.00198 | 6.53E-05 | 34.7871 |
| rs10951371 | G | A | -0.0129 | 0.71228 |  | 1.50E-09 | 0.00214 | 6.86E-05 | 36.537 |
| rs10974435 | C | T | -0.0129 | 0.50506 |  | 3.43E-11 | 0.00194 | 8.28E-05 | 43.9174 |
| rs11001102 | T | C | 0.01805 | 0.16117 | rs7075643 | 7.96E-12 | 0.00264 | 8.81E-05 | 46.7795 |
| rs11007622 | T | C | 0.01454 | 0.2089 |  | 1.20E-09 | 0.00239 | 6.98E-05 | 36.9723 |
| rs11062102 | C | T | 0.02757 | 0.66236 |  | 2.75E-41 | 0.00205 | 0.00034 | 181.166 |
| rs1107366 | G | A | 0.01395 | 0.45283 |  | 9.23E-13 | 0.00195 | 9.64E-05 | 51.0068 |
| rs11102972 | C | T | 0.02142 | 0.22716 |  | 1.86E-20 | 0.00231 | 0.00016 | 85.9433 |
| rs111424562 | G | A | -0.018 | 0.21823 |  | 3.16E-14 | 0.00238 | 0.00011 | 57.6342 |
| rs1114665 | T | C | -0.0119 | 0.45783 |  | 1.08E-09 | 0.00195 | 6.98E-05 | 37.1804 |
| rs11172147 | A | G | -0.0204 | 0.24126 |  | 2.10E-19 | 0.00226 | 0.00015 | 81.1561 |
| rs1118392 | T | C | 0.01305 | 0.67415 |  | 3.22E-10 | 0.00208 | 7.49E-05 | 39.5358 |
| rs112694524 | A | G | 0.03838 | 0.07193 |  | 1.00E-23 | 0.00382 | 0.0002 | 100.844 |
| rs113764569 | T | C | 0.01377 | 0.42672 |  | 6.02E-12 | 0.002 | 9.28E-05 | 47.3266 |
| rs113956264 | T | C | -0.0451 | 0.031005 |  | 1.16E-15 | 0.00563 | 0.00012 | 64.1456 |
| rs11446 | G | A | 0.01337 | 0.63772 |  | 5.90E-11 | 0.00204 | 8.25E-05 | 42.8545 |
| rs11557049 | T | C | 0.03025 | 0.066776 |  | 6.58E-15 | 0.00388 | 0.00011 | 60.7251 |
| rs11616030 | C | A | -0.0264 | 0.087011 |  | 2.13E-14 | 0.00345 | 0.00011 | 58.4108 |
| rs11623989 | C | T | -0.0156 | 0.72336 |  | 7.54E-13 | 0.00217 | 9.68E-05 | 51.4002 |
| rs11634260 | G | T | 0.03911 | 0.26395 | rs10851885 | 3.57E-70 | 0.00221 | 0.00059 | 313.751 |
| rs11670056 | T | C | 0.02745 | 0.074621 |  | 9.48E-14 | 0.00369 | 0.0001 | 55.4754 |
| rs11702255 | A | G | 0.01259 | 0.37526 |  | 7.02E-10 | 0.00204 | 7.43E-05 | 38.0188 |
| rs11706480 | T | C | -0.0309 | 0.32098 |  | 5.51E-50 | 0.00208 | 0.00042 | 221.066 |
| rs11745891 | C | T | 0.03379 | 0.1077 |  | 2.58E-27 | 0.00312 | 0.00022 | 117.234 |
| rs11786896 | T | C | 0.04104 | 0.050308 |  | 5.89E-20 | 0.00449 | 0.00016 | 83.6675 |
| rs11829293 | G | A | 0.01383 | 0.23558 |  | 1.66E-09 | 0.00229 | 6.88E-05 | 36.3314 |
| rs11845147 | A | G | -0.0145 | 0.29813 |  | 7.37E-12 | 0.00212 | 8.82E-05 | 46.9249 |
| rs11856829 | T | C | -0.0158 | 0.43716 | rs4372662 | 4.71E-16 | 0.00195 | 0.00012 | 65.9241 |
| rs11857410 | A | G | -0.0157 | 0.21919 |  | 2.18E-11 | 0.00234 | 8.41E-05 | 44.8039 |
| rs1198975 | A | G | 0.01508 | 0.42103 |  | 1.86E-14 | 0.00197 | 0.00011 | 58.6838 |
| rs12144217 | C | T | -0.0162 | 0.36599 |  | 9.97E-16 | 0.00201 | 0.00012 | 64.4423 |
| rs12159787 | G | A | -0.0322 | 0.092471 |  | 7.20E-22 | 0.00335 | 0.00017 | 92.3754 |
| rs12190605 | T | C | -0.0109 | 0.51731 |  | 2.08E-08 | 0.00195 | 5.94E-05 | 31.4201 |
| rs12211281 | T | C | -0.015 | 0.21569 |  | 1.95E-10 | 0.00236 | 7.62E-05 | 40.516 |
| rs12265758 | G | A | -0.0142 | 0.41222 |  | 7.67E-13 | 0.00199 | 9.81E-05 | 51.3674 |
| rs12434266 | G | A | 0.01318 | 0.60967 |  | 3.28E-11 | 0.00199 | 8.27E-05 | 44.0049 |
| rs12544197 | A | G | 0.01342 | 0.4939 |  | 8.27E-12 | 0.00196 | 9.01E-05 | 46.7034 |
| rs12581220 | T | C | -0.0138 | 0.31684 |  | 5.12E-11 | 0.00209 | 8.19E-05 | 43.1322 |
| rs1258179 | A | G | 0.01455 | 0.49356 |  | 6.77E-14 | 0.00194 | 0.00011 | 56.1361 |
| rs1260326 | C | T | 0.03715 | 0.60687 |  | 2.19E-78 | 0.00198 | 0.00066 | 351.515 |
| rs12652254 | T | G | -0.0181 | 0.20488 |  | 1.43E-13 | 0.00244 | 0.00011 | 54.6703 |
| rs1265842 | C | T | -0.0115 | 0.51912 |  | 3.58E-09 | 0.00195 | 6.60E-05 | 34.8424 |
| rs1269139 | T | C | 0.01513 | 0.2066 |  | 2.78E-10 | 0.0024 | 7.51E-05 | 39.826 |
| rs12791097 | A | G | -0.0156 | 0.17918 |  | 1.05E-09 | 0.00255 | 7.13E-05 | 37.2338 |
| rs12881621 | C | T | -0.0124 | 0.63716 | rs8005804 | 9.81E-10 | 0.00203 | 7.14E-05 | 37.3654 |
| rs12902855 | A | G | 0.02589 | 0.48217 |  | 3.66E-40 | 0.00195 | 0.00033 | 176.028 |
| rs12946188 | A | C | 0.0427 | 0.034452 |  | 8.98E-16 | 0.00531 | 0.00012 | 64.6494 |
| rs13036344 | A | G | -0.0153 | 0.14892 |  | 1.85E-08 | 0.00273 | 5.97E-05 | 31.6502 |
| rs13059257 | T | C | 0.0441 | 0.029335 |  | 1.71E-14 | 0.00575 | 0.00011 | 58.84 |
| rs13064938 | C | T | 0.02049 | 0.61077 | rs13089362 | 5.77E-25 | 0.00199 | 0.0002 | 106.5 |
| rs13159523 | G | A | -0.0159 | 0.47987 |  | 7.82E-16 | 0.00197 | 0.00013 | 64.9202 |
| rs1317983 | C | T | 0.03452 | 0.69386 |  | 8.86E-61 | 0.0021 | 0.00051 | 270.618 |
| rs13240967 | T | C | 0.01825 | 0.69932 |  | 5.23E-18 | 0.00211 | 0.00014 | 74.8005 |
| rs13253155 | T | C | -0.0136 | 0.20313 | rs13252799 | 1.65E-08 | 0.00241 | 6.00E-05 | 31.8743 |
| rs13283908 | C | T | -0.0132 | 0.21147 |  | 2.54E-08 | 0.00237 | 5.83E-05 | 31.032 |
| rs13296330 | C | A | -0.0186 | 0.10784 |  | 4.66E-09 | 0.00317 | 6.66E-05 | 34.3273 |
| rs13325064 | G | A | -0.0136 | 0.47917 |  | 2.19E-12 | 0.00194 | 9.26E-05 | 49.308 |
| rs1371044 | A | G | 0.01255 | 0.23567 |  | 4.43E-08 | 0.00229 | 5.68E-05 | 29.9522 |
| rs1405832 | G | A | -0.0189 | 0.80703 |  | 1.61E-14 | 0.00246 | 0.00011 | 58.9646 |
| rs1440440 | A | C | 0.01381 | 0.4664 |  | 2.03E-12 | 0.00196 | 9.49E-05 | 49.4602 |
| rs1441994 | C | T | 0.01347 | 0.44733 |  | 4.89E-12 | 0.00195 | 8.97E-05 | 47.7353 |
| rs148185902 | A | G | 0.0934 | 0.013807 |  | 1.89E-26 | 0.00878 | 0.00024 | 113.279 |
| rs149822599 | T | C | 0.03628 | 0.023891 |  | 1.84E-08 | 0.00645 | 6.14E-05 | 31.6621 |
| rs151245 | T | G | 0.01606 | 0.60183 |  | 5.45E-16 | 0.00198 | 0.00012 | 65.6329 |
| rs1545837 | C | T | -0.0157 | 0.36201 |  | 8.41E-15 | 0.00202 | 0.00011 | 60.243 |
| rs1547110 | G | T | -0.014 | 0.21578 |  | 3.06E-09 | 0.00237 | 6.66E-05 | 35.1428 |
| rs1548945 | C | T | 0.02442 | 0.58091 |  | 3.41E-35 | 0.00197 | 0.00029 | 153.252 |
| rs1561919 | C | T | -0.0111 | 0.56666 |  | 1.50E-08 | 0.00196 | 6.03E-05 | 32.0496 |
| rs1564348 | C | T | -0.0243 | 0.17068 |  | 3.26E-21 | 0.00257 | 0.00017 | 89.397 |
| rs1609829 | C | T | 0.01437 | 0.70268 |  | 1.66E-11 | 0.00213 | 8.63E-05 | 45.3466 |
| rs166775 | T | C | 0.01305 | 0.72916 |  | 2.00E-09 | 0.00218 | 6.73E-05 | 35.9791 |
| rs16853605 | C | T | 0.01991 | 0.12314 |  | 1.49E-11 | 0.00295 | 8.56E-05 | 45.5497 |
| rs16874052 | G | A | -0.0297 | 0.047215 |  | 7.43E-11 | 0.00457 | 7.95E-05 | 42.4066 |
| rs17050272 | A | G | 0.02195 | 0.40964 |  | 8.02E-29 | 0.00197 | 0.00023 | 124.123 |
| rs17113967 | C | T | 0.02762 | 0.040844 |  | 1.82E-08 | 0.00491 | 5.98E-05 | 31.6836 |
| rs17206784 | T | C | -0.0107 | 0.52401 |  | 4.10E-08 | 0.00195 | 5.68E-05 | 30.1029 |
| rs17420882 | G | T | 0.01826 | 0.27869 |  | 2.78E-17 | 0.00216 | 0.00013 | 71.5018 |
| rs1772976 | T | C | -0.0142 | 0.51618 |  | 2.80E-13 | 0.00194 | 0.0001 | 53.354 |
| rs17786744 | G | A | 0.03273 | 0.41185 |  | 1.38E-61 | 0.00198 | 0.00052 | 274.314 |
| rs1800574 | T | C | 0.04506 | 0.029078 |  | 5.07E-15 | 0.00576 | 0.00011 | 61.2382 |
| rs183342753 | C | T | -0.0717 | 0.007461 |  | 9.54E-10 | 0.01173 | 7.62E-05 | 37.4185 |
| rs184174206 | T | G | -0.0377 | 0.025939 | rs77882218 | 7.82E-10 | 0.00614 | 7.19E-05 | 37.8064 |
| rs184561571 | A | G | -0.0255 | 0.066304 | rs73245338 | 6.38E-11 | 0.00391 | 8.07E-05 | 42.7021 |
| rs1858800 | T | C | -0.0175 | 0.34506 |  | 1.36E-17 | 0.00205 | 0.00014 | 72.9149 |
| rs1887249 | C | T | 0.01907 | 0.63359 |  | 3.46E-21 | 0.00202 | 0.00017 | 89.2761 |
| rs1949651 | T | C | 0.01253 | 0.47116 |  | 1.34E-10 | 0.00195 | 7.82E-05 | 41.2559 |
| rs2014520 | A | G | -0.0135 | 0.41987 |  | 1.05E-11 | 0.00198 | 8.84E-05 | 46.2348 |
| rs2034899 | G | T | 0.01254 | 0.2821 |  | 5.67E-09 | 0.00215 | 6.37E-05 | 33.9465 |
| rs2068888 | A | G | 0.02181 | 0.44889 |  | 3.73E-29 | 0.00195 | 0.00024 | 125.644 |
| rs2075252 | C | T | 0.02191 | 0.75581 |  | 2.54E-22 | 0.00225 | 0.00018 | 94.4457 |
| rs2078348 | T | C | 0.01286 | 0.25444 |  | 7.41E-09 | 0.00222 | 6.27E-05 | 33.4285 |
| rs2106727 | G | A | -0.0178 | 0.63671 |  | 1.12E-18 | 0.00202 | 0.00015 | 77.8444 |
| rs2110381 | T | C | 0.01375 | 0.76795 |  | 3.69E-09 | 0.00233 | 6.74E-05 | 34.7816 |
| rs2145166 | A | G | 0.02046 | 0.13931 |  | 3.19E-13 | 0.00281 | 0.0001 | 53.091 |
| rs2149129 | G | A | 0.02442 | 0.92397 |  | 2.22E-11 | 0.00365 | 8.38E-05 | 44.7702 |
| rs2194079 | G | A | -0.0123 | 0.50979 |  | 2.50E-10 | 0.00194 | 7.56E-05 | 40.034 |
| rs219778 | G | A | -0.0161 | 0.26403 |  | 1.86E-13 | 0.00219 | 0.0001 | 54.1488 |
| rs2219647 | A | G | 0.02216 | 0.22891 |  | 8.63E-22 | 0.00231 | 0.00017 | 92.0277 |
| rs2243315 | G | A | 0.01474 | 0.30127 |  | 2.85E-12 | 0.00211 | 9.15E-05 | 48.7933 |
| rs2255293 | C | T | -0.0141 | 0.79091 |  | 3.53E-09 | 0.00239 | 6.60E-05 | 34.8678 |
| rs2267375 | T | G | 0.01544 | 0.58118 |  | 3.98E-15 | 0.00197 | 0.00012 | 61.717 |
| rs2273571 | T | C | 0.01462 | 0.28275 |  | 1.64E-11 | 0.00217 | 8.67E-05 | 45.3558 |
| rs2279463 | G | A | 0.05003 | 0.14331 |  | 2.88E-73 | 0.00276 | 0.00061 | 327.969 |
| rs2365285 | G | A | 0.02907 | 0.942287 |  | 2.83E-12 | 0.00416 | 9.19E-05 | 48.8071 |
| rs2412608 | T | C | -0.0215 | 0.49893 |  | 3.91E-28 | 0.00195 | 0.00023 | 120.975 |
| rs2433601 | C | T | 0.05472 | 0.36715 | rs1547487 | 5.42E-162 | 0.00202 | 0.00139 | 736.362 |
| rs2453580 | C | T | 0.02693 | 0.38816 |  | 1.12E-41 | 0.00199 | 0.00034 | 182.955 |
| rs2471604 | C | A | 0.03046 | 0.29126 |  | 2.63E-46 | 0.00213 | 0.00038 | 204.2 |
| rs267738 | G | T | -0.0337 | 0.22091 |  | 2.76E-47 | 0.00233 | 0.00039 | 208.663 |
| rs2803956 | G | A | 0.01211 | 0.45403 |  | 5.34E-10 | 0.00195 | 7.27E-05 | 38.553 |
| rs2823139 | A | G | 0.0229 | 0.33843 |  | 8.86E-29 | 0.00206 | 0.00023 | 123.919 |
| rs2834317 | A | G | 0.02761 | 0.15376 |  | 1.77E-24 | 0.0027 | 0.0002 | 104.276 |
| rs28375625 | C | A | -0.0113 | 0.53401 |  | 7.51E-09 | 0.00195 | 6.30E-05 | 33.4004 |
| rs28394165 | C | T | 0.05268 | 0.45707 |  | 5.92E-162 | 0.00194 | 0.00138 | 736.172 |
| rs2863977 | A | G | -0.0149 | 0.58904 |  | 4.77E-14 | 0.00198 | 0.00011 | 56.8248 |
| rs288762 | C | T | 0.02143 | 0.63672 |  | 2.78E-26 | 0.00202 | 0.00021 | 112.518 |
| rs2903386 | C | T | -0.0111 | 0.37053 |  | 3.58E-08 | 0.00201 | 5.71E-05 | 30.3682 |
| rs2935110 | T | G | 0.07826 | 0.009109 |  | 3.23E-12 | 0.01123 | 0.00011 | 48.5523 |
| rs2976181 | T | C | -0.017 | 0.2675 |  | 9.09E-15 | 0.00219 | 0.00011 | 60.0885 |
| rs2992746 | C | T | -0.0157 | 0.15509 |  | 4.38E-09 | 0.00268 | 6.47E-05 | 34.4492 |
| rs3107155 | C | T | -0.0129 | 0.60015 |  | 7.20E-11 | 0.00198 | 7.98E-05 | 42.4695 |
| rs3111258 | G | T | -0.0115 | 0.4349 |  | 4.09E-09 | 0.00196 | 6.53E-05 | 34.5802 |
| rs3220814 | A | G | 0.01542 | 0.62118 | rs7568385 | 2.79E-14 | 0.00203 | 0.00011 | 57.8792 |
| rs327508 | A | G | -0.0155 | 0.21005 |  | 9.98E-11 | 0.0024 | 8.02E-05 | 41.8283 |
| rs34230842 | G | A | 0.01761 | 0.17292 |  | 6.41E-12 | 0.00256 | 8.87E-05 | 47.2033 |
| rs34532102 | T | C | 0.01318 | 0.20656 |  | 4.26E-08 | 0.00241 | 5.69E-05 | 30.0302 |
| rs35411989 | T | C | 0.01306 | 0.49544 |  | 1.77E-11 | 0.00194 | 8.52E-05 | 45.2099 |
| rs36111056 | A | G | 0.02015 | 0.21036 |  | 2.87E-17 | 0.00238 | 0.00013 | 71.4399 |
| rs3744274 | A | C | 0.01216 | 0.6534 |  | 2.36E-09 | 0.00204 | 6.70E-05 | 35.6556 |
| rs3799354 | A | C | -0.0275 | 0.1158 |  | 1.14E-19 | 0.00303 | 0.00015 | 82.3539 |
| rs3818802 | A | G | -0.017 | 0.54355 |  | 2.64E-18 | 0.00195 | 0.00014 | 76.1547 |
| rs3827045 | G | A | 0.01323 | 0.56876 |  | 1.81E-11 | 0.00197 | 8.58E-05 | 45.1676 |
| rs3850625 | A | G | -0.0332 | 0.11856 |  | 1.27E-28 | 0.00299 | 0.00023 | 123.202 |
| rs3918226 | T | C | 0.02 | 0.08145 |  | 2.62E-08 | 0.00359 | 5.99E-05 | 30.9753 |
| rs3925584 | C | T | -0.0321 | 0.4546 |  | 3.03E-61 | 0.00194 | 0.00051 | 272.731 |
| rs41284816 | T | G | 0.06183 | 0.019105 |  | 1.15E-17 | 0.00723 | 0.00014 | 73.2452 |
| rs429358 | C | T | -0.028 | 0.15622 |  | 1.11E-25 | 0.00267 | 0.00021 | 109.763 |
| rs4466248 | C | T | -0.0233 | 0.89388 |  | 1.14E-13 | 0.00314 | 0.0001 | 55.1157 |
| rs4491726 | G | A | 0.02063 | 0.3153 |  | 3.72E-23 | 0.00208 | 0.00018 | 98.2491 |
| rs453528 | C | A | 0.0202 | 0.46811 |  | 1.44E-24 | 0.00197 | 0.0002 | 104.694 |
| rs4575719 | A | C | -0.0139 | 0.60985 |  | 2.58E-12 | 0.00199 | 9.22E-05 | 48.981 |
| rs4649024 | T | C | -0.023 | 0.070611 |  | 1.09E-09 | 0.00378 | 6.97E-05 | 37.1574 |
| rs4666821 | T | G | -0.0131 | 0.51211 |  | 1.60E-11 | 0.00194 | 8.52E-05 | 45.4129 |
| rs4744712 | C | A | -0.0301 | 0.60157 |  | 4.87E-52 | 0.00198 | 0.00043 | 230.474 |
| rs4946930 | A | G | -0.0251 | 0.15626 |  | 5.05E-21 | 0.00267 | 0.00017 | 88.5299 |
| rs4962238 | T | C | -0.0308 | 0.079627 |  | 2.51E-17 | 0.00364 | 0.00014 | 71.706 |
| rs4966019 | T | C | -0.0173 | 0.6405 |  | 1.27E-17 | 0.00202 | 0.00014 | 73.0463 |
| rs5029969 | T | G | 0.01807 | 0.31427 | rs5029970 | 4.49E-18 | 0.00208 | 0.00014 | 75.0995 |
| rs514595 | C | T | -0.0199 | 0.84086 |  | 6.36E-14 | 0.00265 | 0.00011 | 56.2627 |
| rs550057 | T | C | -0.0199 | 0.25475 |  | 3.92E-19 | 0.00223 | 0.00015 | 79.9198 |
| rs55842281 | G | A | -0.0183 | 0.6453 |  | 3.42E-19 | 0.00204 | 0.00015 | 80.1942 |
| rs56255430 | C | A | 0.02465 | 0.084427 |  | 1.70E-12 | 0.00349 | 9.39E-05 | 49.8049 |
| rs56287664 | C | T | 0.01701 | 0.479 |  | 2.03E-18 | 0.00194 | 0.00014 | 76.6763 |
| rs56376587 | C | A | 0.03662 | 0.48835 |  | 3.31E-77 | 0.00197 | 0.00067 | 346.082 |
| rs570801 | G | A | 0.01335 | 0.204 |  | 3.84E-08 | 0.00243 | 5.79E-05 | 30.231 |
| rs6001728 | G | A | 0.01585 | 0.22977 |  | 7.22E-12 | 0.00231 | 8.89E-05 | 46.9699 |
| rs6058108 | G | A | 0.02634 | 0.39455 |  | 3.58E-40 | 0.00198 | 0.00033 | 176.062 |
| rs60865276 | T | G | -0.0156 | 0.36299 |  | 1.92E-14 | 0.00203 | 0.00011 | 58.6184 |
| rs61830291 | C | A | -0.0286 | 0.097064 |  | 2.90E-18 | 0.00328 | 0.00014 | 75.9679 |
| rs62168615 | A | G | 0.01682 | 0.13348 |  | 3.51E-09 | 0.00285 | 6.54E-05 | 34.8809 |
| rs62435145 | T | G | 0.04234 | 0.69191 |  | 3.30E-85 | 0.00216 | 0.00076 | 382.868 |
| rs6497008 | G | A | -0.0137 | 0.81031 |  | 3.10E-08 | 0.00248 | 5.80E-05 | 30.6456 |
| rs6569648 | T | C | -0.0212 | 0.75925 |  | 6.06E-21 | 0.00226 | 0.00016 | 88.1628 |
| rs6679817 | T | C | 0.01254 | 0.36604 |  | 5.06E-10 | 0.00202 | 7.30E-05 | 38.6545 |
| rs6685648 | C | T | 0.01699 | 0.31461 |  | 3.65E-16 | 0.00208 | 0.00012 | 66.4271 |
| rs67648215 | T | C | 0.01922 | 0.15574 |  | 8.11E-13 | 0.00268 | 9.72E-05 | 51.2609 |
| rs6814067 | G | T | 0.01374 | 0.51265 |  | 1.31E-12 | 0.00194 | 9.43E-05 | 50.3231 |
| rs6968534 | G | A | -0.0121 | 0.39506 |  | 1.06E-09 | 0.00199 | 7.01E-05 | 37.2189 |
| rs700750 | A | C | 0.02197 | 0.62888 |  | 5.87E-28 | 0.002 | 0.00023 | 120.166 |
| rs7012814 | A | G | -0.0212 | 0.47506 |  | 1.53E-27 | 0.00195 | 0.00023 | 118.266 |
| rs7036795 | C | T | -0.0156 | 0.19729 |  | 2.64E-10 | 0.00248 | 7.75E-05 | 39.9268 |
| rs7122754 | G | A | 0.01368 | 0.46429 |  | 1.84E-12 | 0.00194 | 9.31E-05 | 49.6459 |
| rs7162890 | A | G | -0.0169 | 0.53065 |  | 2.86E-18 | 0.00194 | 0.00014 | 75.996 |
| rs7214078 | G | A | 0.01068 | 0.54599 |  | 4.22E-08 | 0.00195 | 5.65E-05 | 30.0501 |
| rs7247977 | C | T | -0.033 | 0.3964 |  | 2.52E-62 | 0.00198 | 0.00052 | 277.699 |
| rs72683923 | C | T | -0.048 | 0.020149 |  | 3.54E-12 | 0.0069 | 9.09E-05 | 48.3655 |
| rs72706148 | T | C | 0.05094 | 0.016996 |  | 1.08E-10 | 0.00789 | 8.67E-05 | 41.679 |
| rs72801873 | G | A | 0.05149 | 0.031955 |  | 9.34E-21 | 0.00551 | 0.00016 | 87.3077 |
| rs728538 | G | T | 0.01715 | 0.16687 |  | 5.22E-11 | 0.00261 | 8.18E-05 | 43.097 |
| rs72868875 | C | A | 0.02557 | 0.068747 |  | 2.66E-11 | 0.00384 | 8.37E-05 | 44.4178 |
| rs72904941 | T | C | 0.01871 | 0.11465 |  | 7.65E-10 | 0.00304 | 7.11E-05 | 37.8515 |
| rs72930659 | T | C | -0.0265 | 0.099515 |  | 7.06E-16 | 0.00328 | 0.00013 | 65.1238 |
| rs73036658 | G | A | -0.0172 | 0.3898 |  | 5.78E-18 | 0.00199 | 0.00014 | 74.6022 |
| rs73116951 | T | C | -0.0394 | 0.086031 |  | 7.71E-30 | 0.00347 | 0.00024 | 128.771 |
| rs73119034 | A | G | 0.02079 | 0.12222 | rs73119035 | 4.04E-12 | 0.003 | 9.27E-05 | 48.1083 |
| rs73236061 | G | A | 0.01891 | 0.12654 |  | 1.41E-10 | 0.00295 | 7.91E-05 | 41.1507 |
| rs736820 | A | G | 0.01254 | 0.37691 |  | 3.86E-10 | 0.002 | 7.39E-05 | 39.1796 |
| rs73728279 | T | G | 0.05108 | 0.28248 |  | 2.54E-124 | 0.00215 | 0.00106 | 562.847 |
| rs74465551 | G | A | -0.0195 | 0.090309 |  | 1.13E-08 | 0.00342 | 6.28E-05 | 32.5978 |
| rs74746191 | T | G | -0.016 | 0.40588 | rs9648399 | 6.57E-16 | 0.00198 | 0.00012 | 65.2605 |
| rs7514180 | A | G | -0.0214 | 0.21198 |  | 1.99E-19 | 0.00237 | 0.00015 | 81.2519 |
| rs75432828 | A | G | 0.01628 | 0.31434 |  | 1.26E-14 | 0.00211 | 0.00011 | 59.4494 |
| rs75460349 | C | A | -0.0547 | 0.02367 |  | 2.99E-17 | 0.00647 | 0.00014 | 71.3569 |
| rs7651346 | A | G | 0.02234 | 0.26224 |  | 4.04E-24 | 0.00221 | 0.00019 | 102.638 |
| rs7679542 | G | A | 0.01173 | 0.34902 |  | 8.78E-09 | 0.00204 | 6.25E-05 | 33.0941 |
| rs76871347 | G | A | -0.0161 | 0.32629 |  | 9.58E-14 | 0.00217 | 0.00011 | 55.4589 |
| rs7701668 | A | G | -0.0355 | 0.02345 |  | 3.14E-08 | 0.00642 | 5.78E-05 | 30.6184 |
| rs77375846 | C | T | 0.02259 | 0.13346 |  | 2.36E-15 | 0.00285 | 0.00012 | 62.7414 |
| rs7779637 | G | A | -0.0181 | 0.54751 |  | 1.60E-20 | 0.00195 | 0.00016 | 86.2469 |
| rs77924615 | A | G | -0.0638 | 0.19765 |  | 7.36E-149 | 0.00245 | 0.00129 | 675.856 |
| rs78343805 | T | C | -0.0293 | 0.035235 |  | 3.15E-08 | 0.0053 | 5.85E-05 | 30.614 |
| rs78412834 | A | G | 0.02998 | 0.032801 |  | 3.95E-08 | 0.00546 | 5.70E-05 | 30.1768 |
| rs78444298 | A | G | 0.07968 | 0.019577 |  | 1.13E-29 | 0.00704 | 0.00024 | 128.009 |
| rs791578 | C | T | 0.04636 | 0.89148 |  | 6.21E-50 | 0.00312 | 0.00042 | 220.817 |
| rs796869 | T | G | 0.01073 | 0.46 |  | 3.55E-08 | 0.00195 | 5.72E-05 | 30.3866 |
| rs79820349 | G | A | 0.02629 | 0.047034 |  | 1.60E-08 | 0.00465 | 6.20E-05 | 31.9338 |
| rs80138475 | T | C | -0.0409 | 0.11295 |  | 1.69E-40 | 0.00307 | 0.00034 | 177.555 |
| rs80349960 | C | T | -0.0299 | 0.093814 |  | 6.11E-19 | 0.00336 | 0.00015 | 79.0441 |
| rs807624 | T | G | -0.0243 | 0.35773 |  | 2.59E-33 | 0.00202 | 0.00027 | 144.652 |
| rs8095987 | G | A | 0.01274 | 0.42451 |  | 7.90E-11 | 0.00196 | 7.93E-05 | 42.2845 |
| rs8109944 | T | C | 0.01332 | 0.63342 |  | 3.69E-11 | 0.00201 | 8.24E-05 | 43.7717 |
| rs81205 | C | A | 0.01919 | 0.46203 |  | 3.68E-22 | 0.00198 | 0.00018 | 93.7063 |
| rs847161 | G | A | 0.02022 | 0.78168 |  | 1.16E-17 | 0.00236 | 0.00014 | 73.2342 |
| rs858193 | G | A | 0.01432 | 0.46837 |  | 2.08E-13 | 0.00195 | 0.0001 | 53.9318 |
| rs882759 | G | A | -0.0178 | 0.36028 |  | 1.51E-18 | 0.00202 | 0.00015 | 77.2476 |
| rs901107 | A | G | 0.01374 | 0.38745 |  | 7.32E-12 | 0.00201 | 8.96E-05 | 46.9405 |
| rs9264791 | C | T | -0.0187 | 0.21425 |  | 2.17E-15 | 0.00236 | 0.00012 | 62.905 |
| rs9318186 | G | A | 0.01267 | 0.55652 |  | 1.29E-10 | 0.00197 | 7.92E-05 | 41.3312 |
| rs9385649 | A | G | -0.0175 | 0.3203 |  | 3.23E-17 | 0.00207 | 0.00013 | 71.2049 |
| rs940131 | C | T | 0.01696 | 0.47183 |  | 7.22E-18 | 0.00197 | 0.00014 | 74.16 |
| rs9419939 | A | G | -0.0219 | 0.17648 |  | 7.30E-18 | 0.00255 | 0.00014 | 74.1452 |
| rs9465741 | A | C | -0.0158 | 0.55154 |  | 8.34E-16 | 0.00196 | 0.00012 | 64.7943 |
| rs9474334 | G | A | 0.01369 | 0.57166 |  | 3.07E-12 | 0.00196 | 9.18E-05 | 48.6475 |
| rs948494 | A | G | 0.02116 | 0.34218 |  | 3.14E-25 | 0.00204 | 0.0002 | 107.715 |
| rs952359 | A | G | 0.01636 | 0.33744 |  | 1.51E-15 | 0.00205 | 0.00012 | 63.6214 |
| rs9529913 | T | C | 0.01711 | 0.60193 |  | 6.80E-18 | 0.00198 | 0.00014 | 74.2774 |
| rs9691744 | T | G | -0.0157 | 0.34434 |  | 1.66E-14 | 0.00205 | 0.00011 | 58.9044 |
| rs9818740 | A | G | -0.0169 | 0.27503 |  | 6.46E-15 | 0.00217 | 0.00011 | 60.7572 |
| rs9823161 | G | A | 0.01614 | 0.30629 |  | 2.39E-14 | 0.00212 | 0.00011 | 58.1896 |
| rs9895661 | T | C | -0.0542 | 0.83112 |  | 2.67E-97 | 0.00259 | 0.00082 | 438.464 |
| rs9905432 | A | C | -0.036 | 0.25262 |  | 1.28E-58 | 0.00223 | 0.00049 | 260.675 |
| rs9960465 | T | C | -0.0228 | 0.2081 |  | 1.52E-21 | 0.00239 | 0.00017 | 90.8964 |
