# Supplementary material for: Mendelian randomization study supports the causal association between serum cystatin C and risk of diabetic nephropathy
Source: Front Endocrinol (Lausanne). 2022 Nov 17;13:1043174. doi: 10.3389/fendo.2022.1043174 (PMC9724588; doi:10.3389/fendo.2022.1043174)
Supplement: Supplementary file 5 [file Table_5.docx]

**Supplementary Table 5:** Instrumental variables of KIM-1. SNP, the rsID of genetic variants; A1, the effect allele; A2, the other allele; Beta, the effect size of A1 on the exposure; Se, the standard error of beta; Proxy, the proxy SNP in the outcome; P, the p-value of beta; R2, the proportion of variance explained by each SNP; F, the F statistic

| SNP | A1 | A2 | Beta | EAF | Se | P | R2 | F | SNP |
| --- | --- | --- | --- | --- | --- | --- | --- | --- | --- |
| rs12952262 | T | C | -0.0897 | 0.1648 | 0.0144 | 5.02E-10 | 0.00221 | 38.8025 | rs12952262 |
| rs1345616 | A | G | -0.4442 | 0.2444 | 0.0116 | 1.00E-200 | 0.07288 | 1466.36 | rs1345616 |
| rs140138767 | A | G | 0.2728 | 0.0176 | 0.0499 | 4.54E-08 | 0.00257 | 29.8874 | rs140138767 |
| rs140702195 | T | C | -1.2026 | 0.0122 | 0.0542 | 3.99E-109 | 0.03486 | 492.316 | rs140702195 |
| rs17053768 | G | A | -0.1675 | 0.042 | 0.0266 | 2.98E-10 | 0.00226 | 39.6521 | rs17053768 |
| rs191192726 | T | C | 0.3155 | 0.0181 | 0.0408 | 1.11E-14 | 0.00354 | 59.7969 | rs191192726 |
| rs2991996 | A | G | 0.0753 | 0.4294 | 0.0105 | 7.31E-13 | 0.00278 | 51.4294 | rs2991996 |
| rs4148005 | G | T | 0.0817 | 0.3127 | 0.0111 | 1.85E-13 | 0.00287 | 54.1749 | rs4148005 |
| rs76749041 | T | C | 0.2622 | 0.0375 | 0.0285 | 3.74E-20 | 0.00496 | 84.64 | rs76749041 |
| rs77924615 | A | G | -0.1383 | 0.198 | 0.0136 | 2.29E-24 | 0.00607 | 103.411 | rs77924615 |
| rs8036675 | A | C | -0.06 | 0.4185 | 0.0107 | 2.31E-08 | 0.00175 | 31.4438 | rs8036675 |
