# Supplementary material for: Mendelian randomization study supports the causal association between serum cystatin C and risk of diabetic nephropathy
Source: Front Endocrinol (Lausanne). 2022 Nov 17;13:1043174. doi: 10.3389/fendo.2022.1043174 (PMC9724588; doi:10.3389/fendo.2022.1043174)
Supplement: Supplementary file 6 [file Table_6.docx]

**Supplementary Table 6**: Instrumental variables of GDF-15. SNP, the rsID of genetic variants; A1, the effect allele; A2, the other allele; Beta, the effect size of A1 on the exposure; Se, the standard error of beta; Proxy, the proxy SNP in the outcome; P, the p-value of beta; R2, the proportion of variance explained by each SNP; F, the F statistic

| SNP | A1 | A2 | Beta | EAF | Proxy | P | Se | R2 | F |
| --- | --- | --- | --- | --- | --- | --- | --- | --- | --- |
| rs113090220 | T | G | -0.0869 | 0.0676 |  | 0.0186 | 3.00E-06 | 0.00095 | 21.828 |
| rs115425981 | T | C | 0.1793 | 0.0236 |  | 0.0387 | 3.53E-06 | 0.00148 | 21.4654 |
| rs1227734 | T | C | 0.3689 | 0.1362 |  | 0.013 | 9.93E-177 | 0.03202 | 805.25 |
| rs13440344 | A | C | -0.0529 | 0.2546 |  | 0.0109 | 1.31E-06 | 0.00106 | 23.5537 |
| rs139418 | C | A | -0.0434 | 0.4483 |  | 0.0096 | 5.68E-06 | 0.00093 | 20.4379 |
| rs139519459 | A | G | 0.3139 | 0.0115 |  | 0.0603 | 1.94E-07 | 0.00224 | 27.0987 |
| rs144235567 | A | G | 0.1859 | 0.0258 |  | 0.0384 | 1.27E-06 | 0.00174 | 23.4367 |
| rs148458930 | A | G | -0.2078 | 0.0196 |  | 0.0461 | 6.56E-06 | 0.00166 | 20.3184 |
| rs164514 | G | A | -0.0528 | 0.3321 |  | 0.0108 | 9.70E-07 | 0.00124 | 23.9012 |
| rs2188939 | A | G | 0.0454 | 0.6803 |  | 0.01 | 5.25E-06 | 0.0009 | 20.6116 |
| rs241816 | T | C | 0.0494 | 0.6625 |  | 0.0104 | 2.17E-06 | 0.00109 | 22.5625 |
| rs35941406 | T | G | -0.0458 | 0.4369 |  | 0.0098 | 2.91E-06 | 0.00103 | 21.8413 |
| rs3924076 | A | G | -0.0508 | 0.2668 |  | 0.0114 | 7.84E-06 | 0.00101 | 19.8572 |
| rs58355095 | G | T | 0.082 | 0.3555 | rs12415666 | 0.0178 | 3.94E-06 | 0.00308 | 21.2221 |
| rs62355379 | T | G | -0.0484 | 0.6381 |  | 0.0106 | 5.29E-06 | 0.00108 | 20.8487 |
| rs62530603 | C | T | 0.0958 | 0.0987 |  | 0.0208 | 4.33E-06 | 0.00163 | 21.2131 |
| rs6698967 | G | A | 0.1526 | 0.0434 |  | 0.0316 | 1.35E-06 | 0.00193 | 23.3203 |
| rs76857789 | T | C | -0.1567 | 0.0342 |  | 0.0347 | 6.16E-06 | 0.00162 | 20.3929 |
