# Supplementary material for: Mendelian randomization study supports the causal association between serum cystatin C and risk of diabetic nephropathy
Source: Front Endocrinol (Lausanne). 2022 Nov 17;13:1043174. doi: 10.3389/fendo.2022.1043174 (PMC9724588; doi:10.3389/fendo.2022.1043174)
Supplement: Supplementary file 7 [file Table_7.docx]

**Supplementary Table 7**: Sensitive test of six serum biomarkers. Q IVW, Q statistic of calculated in inverse-variance weighted method; df, degree of freedom; P1, p-value of Q statistic; P2, p-value of the MR-Egger Intercept

| Exposure | Q IVW | df | P1 | MR-Egger Intercept | P2 |
| --- | --- | --- | --- | --- | --- |
| Cystatin C | 292.8915 | 233 | 0.004698167 | 0.005605858 | 0.059949 |
| Cystatin C-corrected | 238.4858 | 228 | 0.3033403 | 0.002796566 | 0.533065 |
| TBL | 125.989 | 98 | 0.02991721 | -0.002937962 | 0.453178 |
| Urate | 226.3966 | 187 | 0.02598638 | 0.001947228 | 0.560043 |
| Creatinine | 306.5286 | 242 | 0.003105843 | -0.003633676 | 0.4750322 |
| KIM-1 | 24.47973 | 10 | 0.006423946 | 0.04051547 | 0.081842 |
| GDF-15 | 14.9882 | 17 | 0.5963315 | -0.000510765 | 0.967981 |
