# Supplementary figures and images for: Mendelian randomization study supports the causal association between serum cystatin C and risk of diabetic nephropathy
Source: Front Endocrinol (Lausanne). 2022 Nov 17;13:1043174. doi: 10.3389/fendo.2022.1043174 (PMC9724588; doi:10.3389/fendo.2022.1043174)

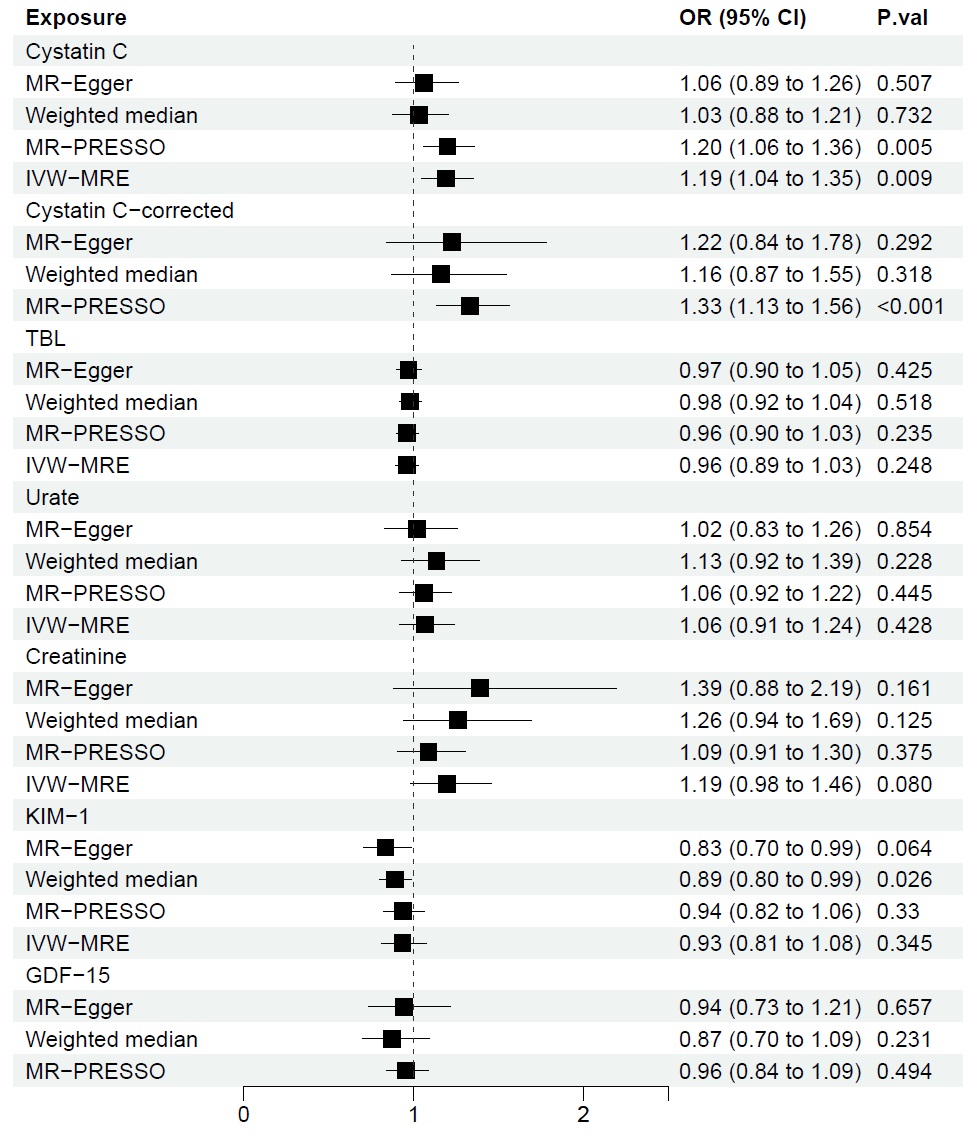

Supplement: Supplementary file 8 [file Image_1.jpeg]

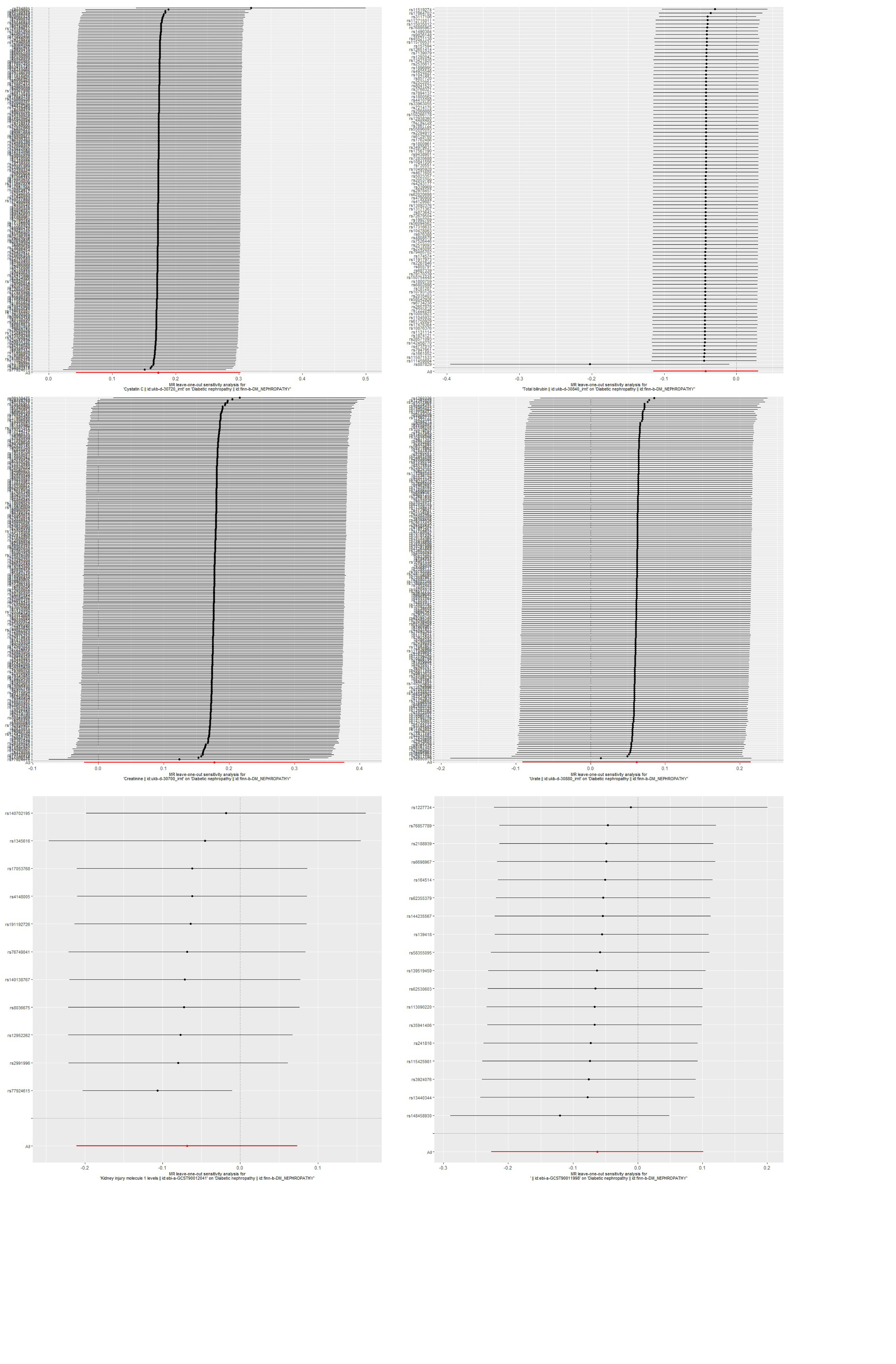

Supplement: Supplementary file 9 [file Image_2.jpeg]
